# Local vs. systemic administration of bisphosphonates in rat cleft bone graft: A comparative study

**DOI:** 10.1371/journal.pone.0190901

**Published:** 2018-01-05

**Authors:** Christine Hong, Alison Quach, Lawrence Lin, Jeffrey Olson, Taewoo Kwon, Olga Bezouglaia, Jaime Tran, Michael Hoang, Kimberly Bui, Reuben H. Kim, Sotirios Tetradis

**Affiliations:** 1 Section of Orthodontics, Division of Growth and Development, UCLA School of Dentistry, Los Angeles, California, United States of America; 2 UCLA School of Dentistry, Los Angeles, California, United States of America; 3 Section of Oral and Maxillofacial Radiology, Division of Diagnostic and Surgical Sciences, UCLA, Los Angeles, California, United States of America; 4 Section of Restorative Dentistry, Division of Constitutive and Regenerative Sciences, UCLA, Los Angeles, California, United States of America; Medical University of South Carolina, UNITED STATES

## Abstract

A majority of patients with orofacial cleft deformity requires cleft repair through a bone graft. However, elevated amount of bone resorption and subsequent bone graft failure remains a significant clinical challenge. Bisphosphonates (BPs), a class of anti-resorptive drugs, may offer great promise in enhancing the clinical success of bone grafting. In this study, we compared the effects of systemic and local delivery of BPs in an intraoral bone graft model in rats. We randomly divided 34 female 20-week-old Fischer F344 Inbred rats into four groups to repair an intraoral critical-sized defect (CSD): (1) Control: CSD without graft (n = 4); (2) Graft/Saline: bone graft with systemic administration of saline 1 week post-operatively (n = 10); (3) Graft/Systemic: bone graft with systemic administration of zoledronic acid 1 week post-operatively (n = 10); and (4) Graft/Local: bone graft pre-treated with zoledronic acid (n = 10). At 6-weeks post-operatively, microCT volumetric analysis showed a significant increase in bone fraction volume (BV/TV) in the Graft/Systemic (62.99 ±14.31%) and Graft/Local (69.35 ±13.18%) groups compared to the Graft/Saline (39.18±10.18%). Similarly, histological analysis demonstrated a significant increase in bone volume in the Graft/Systemic (78.76 ±18.00%) and Graft/Local (89.95 ±4.93%) groups compared to the Graft/Saline (19.74±18.89%). The local delivery approach resulted in the clinical success of bone grafts, with reduced graft resorption and enhanced osteogenesis and bony integration with defect margins while avoiding the effects of BPs on peripheral osteoclastic function. In addition, local delivery of BPs may be superior to systemic delivery with its ease of procedure as it involves simple soaking of bone graft materials in BP solution prior to graft placement into the defect. This new approach may provide convenient and promising clinical applications towards effectively managing cleft patients.

## Introduction

Orofacial cleft anomalies are the most common craniofacial congenital aberrations to occur worldwide [1] and these clefts may manifest as part of a syndrome or more commonly, as isolated cases [2]. The etiology of orofacial cleft, however, is complex and thought to be multifactorial with both genetic and environmental components [3].

A multitude of clinical problems are associated with cleft patients, including deficient facial growth, malocclusion, and respiratory, feeding, and speech complications [4], requiring a comprehensive and multi-disciplinary approach for their care. Bone graft surgery is an essential step in the comprehensive treatment of cleft patients. Bone grafting provides: 1) stabilization of the maxilla, thereby helping maintain palatal width and preventing collapse following expansion, 2) a scaffold for tooth eruption or future implant placement, 3) effective closure of oronasal fistulas, 4) support for the alar base of the nose and lip, and 5) improvement of esthetic results and overall facial symmetry [5,6,7]. However, insufficient bone volume in the cleft region due to a high amount of bone resorption has been a major clinical challenge in cleft patient care.

Bisphosphonates (BPs), anti-resorptive drugs, have been universally used to treat various skeletal and metabolic conditions characterized by enhanced osteoclast-mediated bone resorption, including osteoporosis and cancer. They are also safely utilized in pediatric patients, most notably for osteogenesis imperfecta [8]. With respect to bone grafting, both systemic and local applications of BPs have been shown to be effective in inhibiting bone resorption [9–11] as well as in enhancing bone formation [10,12]. While the exact differences in efficacy of local versus systemic delivery of BPs remain elusive, local delivery is expected to be a superior method of delivery in minimizing undesired systemic side effects. Local delivery of BPs can readily be accomplished by immersing the bone graft material in the BP solution [9,13]. As a result, BP delivery can be targeted to graft regions in high concentrations with little effect elsewhere. Indeed, McKenzie et al. showed that local elution of BP from porous implants had minimal systemic BP distribution [14]. In a rat model, local BP treatment improved implant fixation without inducing bisphosphonate-related osteonecrosis of the jaw (BRONJ)-like lesions, one of the known extreme side effects of BP [15]. However, the clinical efficacy of local delivery remains to be determined in an intraoral cleft bone-grafting model.

In the present study, we assessed the effect of zoledronic acid, a highly potent BP, on bone grafting outcomes and compared the mode of delivery between local and systemic administration in an intraoral bone graft rat model. We hypothesized that local BP delivery would exhibit greater bone volume with reduced resorption as compared with systemic delivery. For comparison purposes, volumetric analysis of bone volume fraction (BV/TV), bone area/total tissue area (MA/TA), bone graft/total tissue area (BG/TA), bone mineral density (BMD), histological examination, TRAP staining to quantify osteoclasts, and assay of TRAP-5b levels were performed.

## Materials and methods

### Ethics statement

All experimental procedures involving animals were conducted according to the UCLA Institutional Animal Care and Use Committee guidelines upon approval (2014-047-03) and National Centre for the Replacement, Refinement, and Reduction of Animals in Research guidelines.

### Animals and bisphosphonate

A total of forty 20-week-old female Fischer F344 Inbred rats with an average weight of 180 g ± 15.1g were purchased from a commercial company (Charles River Laboratories, Wilmington, MA). The rats were housed in light and temperature-controlled facilities, and given food and water *ad libitum*. Zoledronic Acid, a nitrogen-containing bisphosphonate, was purchased from a commercial company (Novartis, Hanover, NJ).

### Experimental methods and design

**Fig 1:**
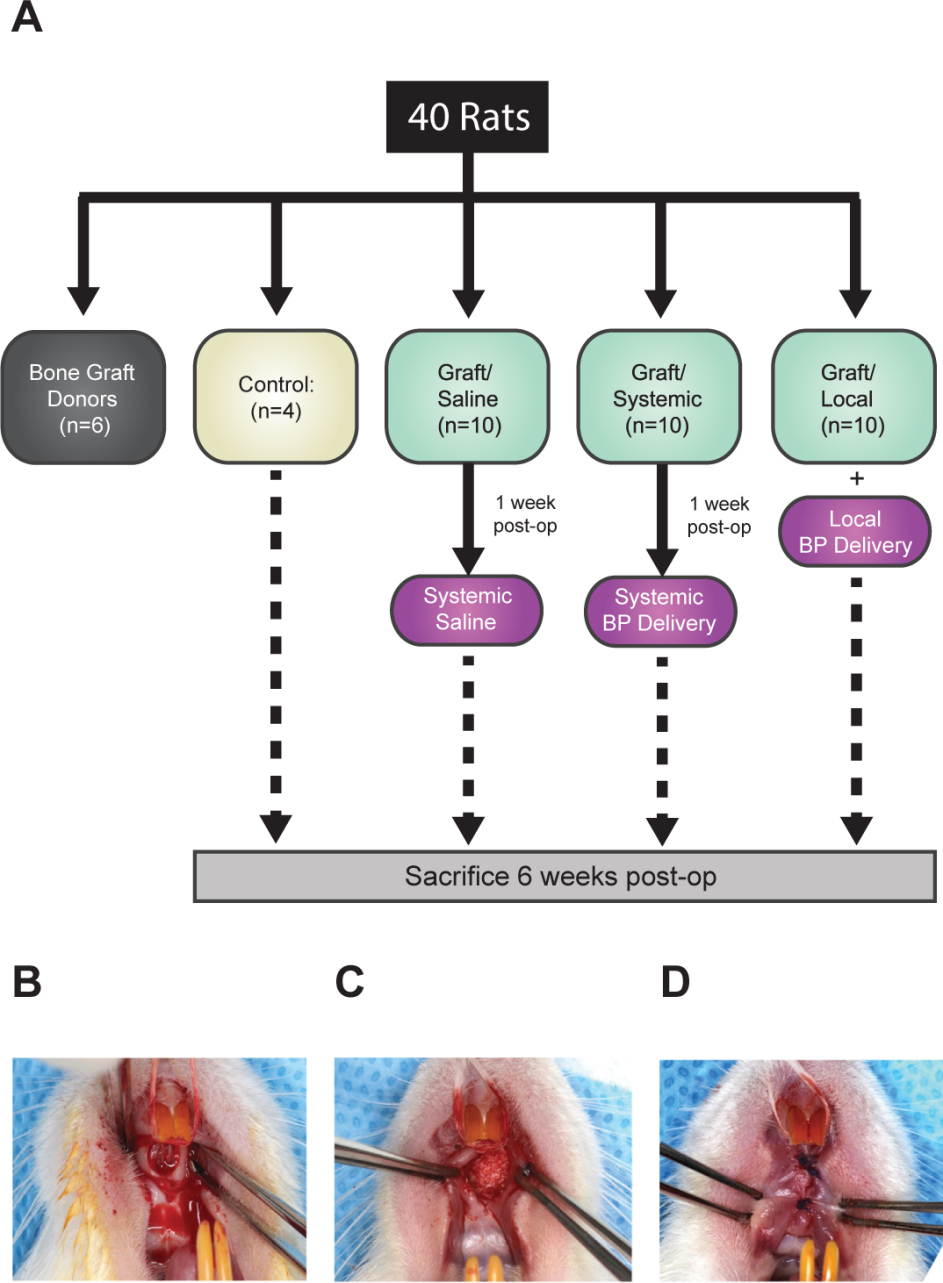
Experimental design and palatal defect/bone grafting animal model. (A) Thirty-four Fischer F344 Inbred rats were divided into four groups: (1) Control: The defect was created but no bone graft was placed; (2) Graft/Saline: Bone graft with systemic saline injection; (3) Graft/Systemic: Bone graft with systemic BP injection; (4) Graft/Local: Bone graft with local BP delivery. (B) A longitudinal mucosal incision made down the middle of the palate and a surgically created 3 mm defect using a slow-speed handpiece and trephine bur. (C) Placement of bone graft particles from isograft donors. (D) Palatal soft tissue closure with simple, interrupted sutures.

Based on our previous study that investigated the effects of systemic BP administration on the success of bone grafts in rats using the same intraoral CSD as in this study, it was determined that 8 rats per experimental group using sample size calculation, n = [z_1-α/2_ + z_1-β_]^2^[σ_1_^2^ + σ_2_^2^] / [μ_1_ –μ_2_]^2^) (n = sample size, z = standard normal distribution percentile, α = type I error, β = type II error, σ = population standard devision, μ = population mean) [16]. Type I error was set at 5% and type II error was set at 20%. The power analysis was performed by UCLA Biostatistics Department. After 2 weeks of acclimatization and confirmation of good health, animals were randomly divided into four groups: (1) Control: defect left empty without graft (n = 4); (2) Graft/Saline: graft with systemic saline injection (n = 10); (3) Graft/Systemic: graft with systemic BP injection (n = 10); (4) Graft/Local: graft pre-treated with BP prior to placement (n = 10). Saline and BP injections were performed at 1-week postoperatively. The group assignment and experimental timeline are illustrated in Fig 1A. All surgeries were performed in the morning at the UCLA vivarium and animals were euthanized 6 weeks post-operatively with CO_2_ asphyxiation.

### Bone graft harvest

An incision was made on the lower back and skin was reflected on donor rats. Blunt dissection to gain access to the pelvic bone was performed. The corticocancellous bone from the iliac crest and femur was harvested bilaterally from 6 donor rats and cartilaginous tissues were removed. A bone mill (G. Hartzell & Son, Concord, CA) was used to reduce the bone size to consistent and uniform particles. The bone particles were then placed on ice for immediate use.

### Creation of intraoral critical-sized defect (CSD) and placement of the bone graft

General anesthesia was initially achieved with isoflurane (4–5%) followed by a combination of intramuscular (IM) ketamine (40 mg/kg) and xylazine (10 mg/kg). Buprenorphine (0.01–0.05 mg/kg) was given subcutaneously after the induction of anesthesia. With animals in supine position, a 1 cm longitudinal mucosal incision was made from behind the maxillary incisors down the middle of the palate. The periosteum was elevated to expose palatal bone. Mid-palatal CSD (3 mm in diameter) was created with a trephine bur under constant irrigation and with care to avoid injury to the adjacent bone (Fig 1B). The harvested cancellous isograft was placed into the defect and packed slightly beyond the margins of the defect (Fig 1C). The oral mucosa was re-approximated with multiple interrupted sutures (Fig 1D). Beginning the day before surgery and for two weeks after surgery, trimethoprimsulfamethoxazole (TMS) was placed in the drinking water (5 ml TMS/500 ml water) to prevent infection.

### BP delivery

Graft/Saline and Graft/Systemic rats received a single subcutaneous injection of saline or zoledronic acid (0.1 mg/kg) one-week post-operatively as previously described [16]. For Graft/Local rats, bone graft particles were immersed in BP solution (0.005 mg/ml) for three minutes, followed by three one-minute washes with gentle agitation to remove excess BP as previously described [10].

### MicroCT analysis

Rat maxillae were fixed with 4% (w/v) paraformaldehyde in 0.1 M phosphate-buffered saline (PBS) for 48 hours and transferred and stored in 70% ethanol. Samples were scanned with high-resolution microCT (SkyScan 1172, SkyScan N.V., Belgium). 3D images were reconstructed using image reconstruction software (NRecon, SkyScan N.V., Belgium) and visualized with orthodontic imaging software (Dolphin Imaging V11.7, Chatsworth, CA). 3D volumetric analysis of the defect was completed with microCT analysis software (CTAn software, SkyScan N.V., Belgium), repeated at two separate times by a single, trained operator. A cylindrical Volume of Interest (VOI) was demarcated by the defect edges in the transaxial view and extended the full depth from palatal surface to the floor of the defect at nasal mucosa. Bone volume (BV), tissue volume (TV), as well as bone mineral density (BMD) were measured and bone volume fraction (BV/TV) was calculated (n = 8 for all three groups).

### Histological analysis

The maxillary specimens were decalcified in 14.5% ethylenediaminetetracetic acid (EDTA 0.1 M, pH = 7.4) solution for 28 days. Samples were washed and then dehydrated in 70% ethanol. Cuts were made coronally through the center of the defect and both anterior and posterior sections were embedded in paraffin. The mineralized area/total tissue area (MA/TA) and bone graft/total tissue area (BG/TA) were calculated with image analysis software (Advanced SPOT 4.6, Macomb County, MI) (n = 5 for all three groups).

### TRAP staining

Decalcified tissues were processed to obtain formalin-fixed paraffin-embedded (FFPE) blocks and 5 μm sections were prepared. The sections were then de-paraffinized at 60°C for 30 min, then rehydrated through xylene and graded ethanol. Slides were incubated for 1 hour at 37°C with TRAP staining solution, according to manufacturer's protocol (Sigma-Aldrich, Inc., St. Louis, MO). The slides were counterstained with hematoxylin solution for 2 min and mounted with aqueous mounting solution. For quantification, osteoclasts were defined as multinucleated (≥ 3 nuclei) TRAP-positive cells. Osteoclasts were counted on the external surfaces of the bone using the surgical defect as the field of view. A single operator at two separate time points performed the quantification using image processing program designed for scientific multidimensional images (ImageJ software, National Institutes of Health, Bethesda, MD) (n = 5 for all three groups).

### TRAP-5b ELISA assay

To study the systemic osteoclastic activity, 300 μl of peripheral blood was collected from each animal at 2 and 6 weeks post-operatively. Samples were centrifuged at 3,000 rpm for 15 min at 4°C to obtain blood serum and supernatants were collected. Serum TRAP-5b levels were quantified using RatTRAP-5b ELISA kit according to the manufacturer’s protocol (Immunodiagnostic Systems, Gaithersburg, MD).

### Statistical analysis

Statistical analysis was performed using scientific 2D graphing and statistics software (GraphPad Prism 6, GraphPad Inc., San Diego, CA). Shapiro-Wilk test was carried out to test for normal distribution and normality was accepted. One-way ANOVA was employed for multiple comparisons between the three groups followed by Tukey’s post-hoc methods to adjust for Type I errors. Values of p ≤ 0.05 were considered to be statistically significant.

## Results

### Effect of BP on bone grafting

**Fig 2:**
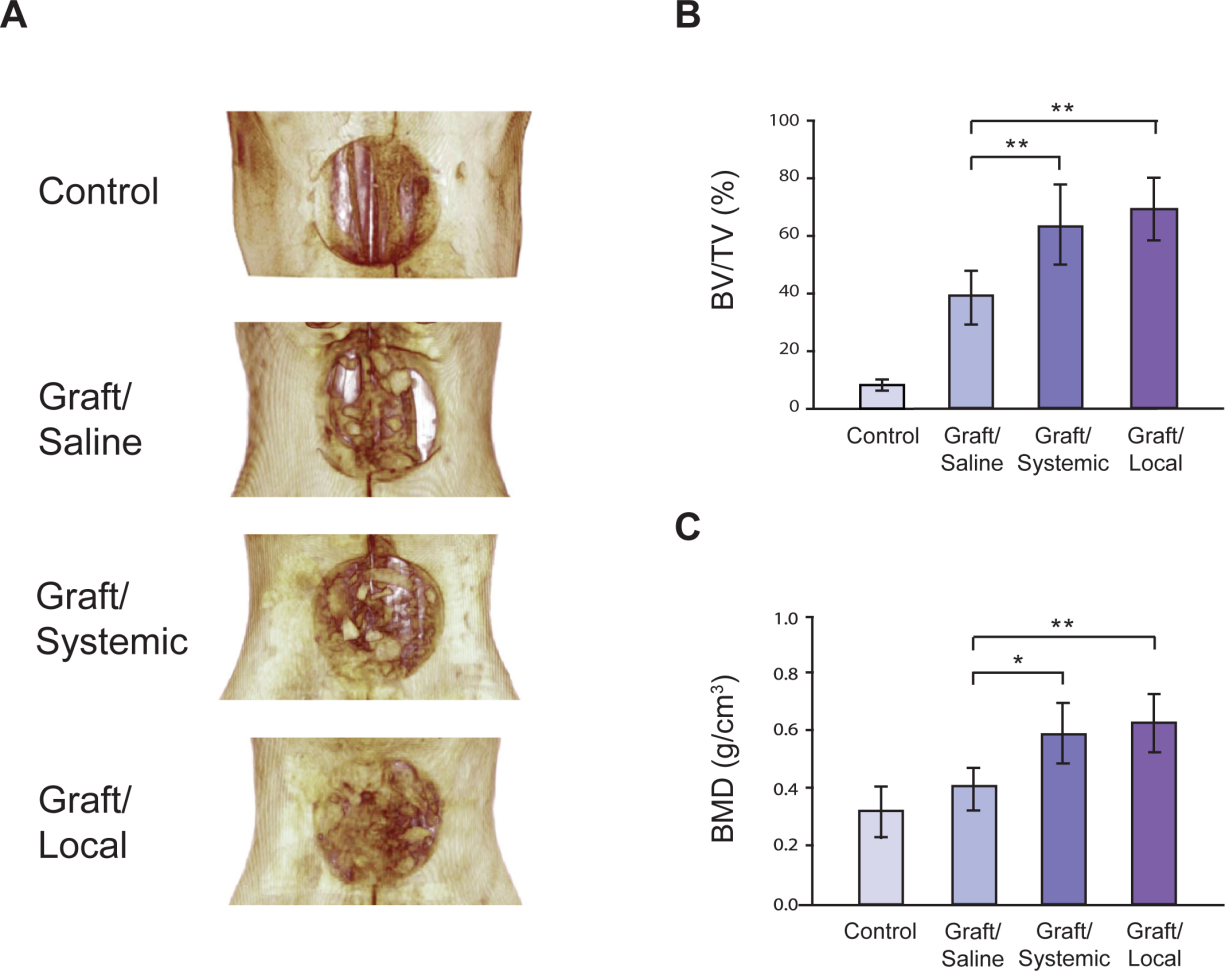
MicroCT images and 3D volumetric analysis. (A) 3D image reconstruction of palatal defect and grafting six weeks after surgery. Quantification of bone volume by volumetric analysis of microCT images for the control and three experimental groups (n = 8); (B) bone volume fraction (BV/TV) and (C) bone mineral density (BMD). * = p < 0.05, ** = p < 0.01.

Postoperative healing was uneventful in all animals and soft tissue healing was complete by one-week post-surgery. Complications such as premature exposure of the augmented sites or infections were not observed throughout the study period. To evaluate bone graft retention and bone regeneration in the intraoral CSD, high-resolution microCT was used for 3D qualitative and quantitative analysis. 3D reconstructed images of the defect area confirmed that the Control group defect was a non-healing CSD (BV/TV = 8.71±2.21%). In both BP-treated groups, however, the bone graft was incorporated with the existing palatal bone defect margins, suggesting clinical success of the grafting procedure (Fig 2A). This defect integration was most evident in the Graft/Local group. Volumetric analysis showed a significant increase (~two-fold) in BV/TV in the Graft/Systemic (62.99 ±14.31%) and Graft/Local (69.35 ±13.18%) groups compared to the Graft/Saline (39.18±10.18%) (Fig 2B). Although the Graft/Local defects displayed the greatest bone volume, it was not significantly greater than the Graft/Systemic defects. Bone mineral density (BMD) analysis also showed a significant increase in Graft/Systemic (0.59 ± 0.12g/cm^3^) and Graft/Local (0.63 ± 0.12g/cm^3^) groups compared to the Graft/Saline group (0.41 ± 0.09g/cm^3^) (Fig 2C). Similarly, the Graft/Local group demonstrated the highest BMD without statistical significance compared to the Graft/Systemic.

**Fig 3:**
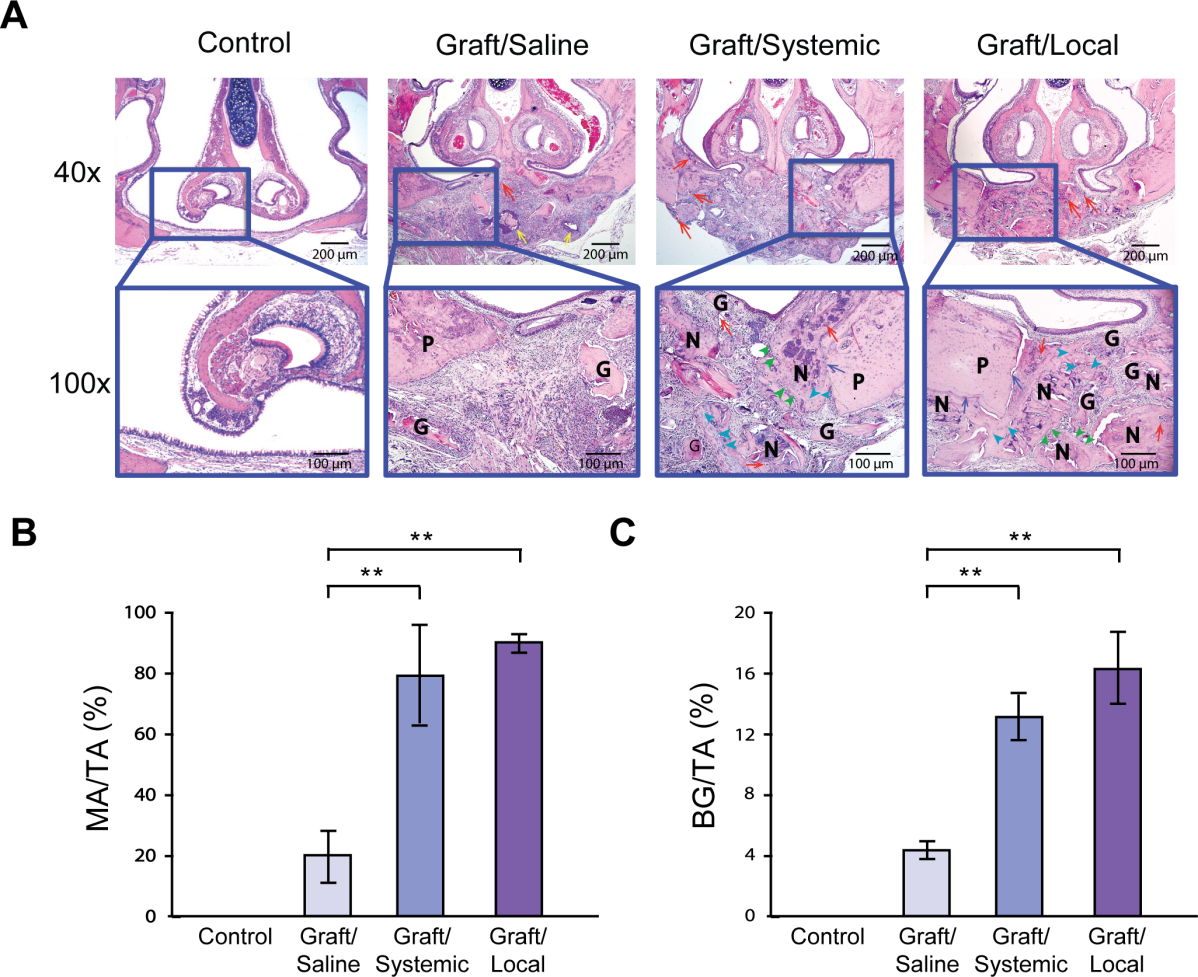
Histomorphometric analysis. (A) H&E stained coronal sections at 40x and 100x magnifications of all four groups of rats **G**: graft, **P**: palatal bone, **N**: new bone, red arrows: blood vessels around bone graft particles, yellow arrows: acute inflammation, blue arrows: bone integration with defect margins, green arrowheads: osteoblasts, light blue arrowheads: osteocytes. (B) Quantifications of bone area/total tissue area (MA/TA) for each group using an image analysis software (Advanced SPOT 4.6, Macomb County, MI). (C) Quantification of bone graft/total tissue area (BG/TA) for each group using Advanced SPOT 4.6 software. * = p < 0.05, ** = p < 0.01.

H&E histological analysis of the defect confirmed enhanced bone graft retention and bone regeneration in BP-treated groups (Fig 3A). In the BP-treated groups, the proliferation of blood vessels (Fig 3A, red arrows) suggested that revascularization of the graft, an essential step in graft survival, had occurred. Furthermore, an increase in immature woven bone, osteoblasts, and osteocytes (Fig 3A, green and light blue arrowheads) indicated active bone remodeling and new bone formation. Bony bridging (Fig 3A, blue arrows) was observed between the graft and defect margins indicating clinical success of bone grafting procedure. The Graft/Local group displayed clear bone formation in the defect region. Conversely, the Graft/Saline group lacked new bone formation with graft resorption. Prominent inflammatory infiltrate (Fig 3A, yellow arrows) was noted in this group. In addition, while existing palatal bone defect integration (Fig 3A, blue arrows) was evident in BP-treated groups, most of the defect margins in the Graft/Saline group demonstrated non-union between palatal bone and bone graft.

Quantitative analysis of bone area/total tissue area (MA/TA) for the Graft/Saline group (19.74±18.89%) contrasted sharply with the four-fold increase in mineralized areas observed in the Graft/Systemic (78.76±18.00%) and Graft/Local (89.95±4.932%) groups (Fig 3B). The Graft/Local group had the greatest bone area/total tissue area without statistical difference from the Graft/Systemic group. In order to examine the anti-resorptive effects of BPs on bone graft, we performed quantitative analysis of remaining bone graft materials in the defect. The analysis of bone graft/total tissue area (BG/TA) showed a ~three-fold increase in bone graft retention in the Graft/Systemic (13.45±2.54%) and Graft/Local (16.95±3.41%) groups (Fig 3C) versus Graft/Saline (5.11±1.43%) Taken together, our data suggest that both systemic and local BP administration enhanced CSD bone grafting outcomes by reducing the resorption of bone graft and increasing bone area relative to total tissue area.

### Effect of bisphosphonate on osteoclast activity

**Fig 4:**
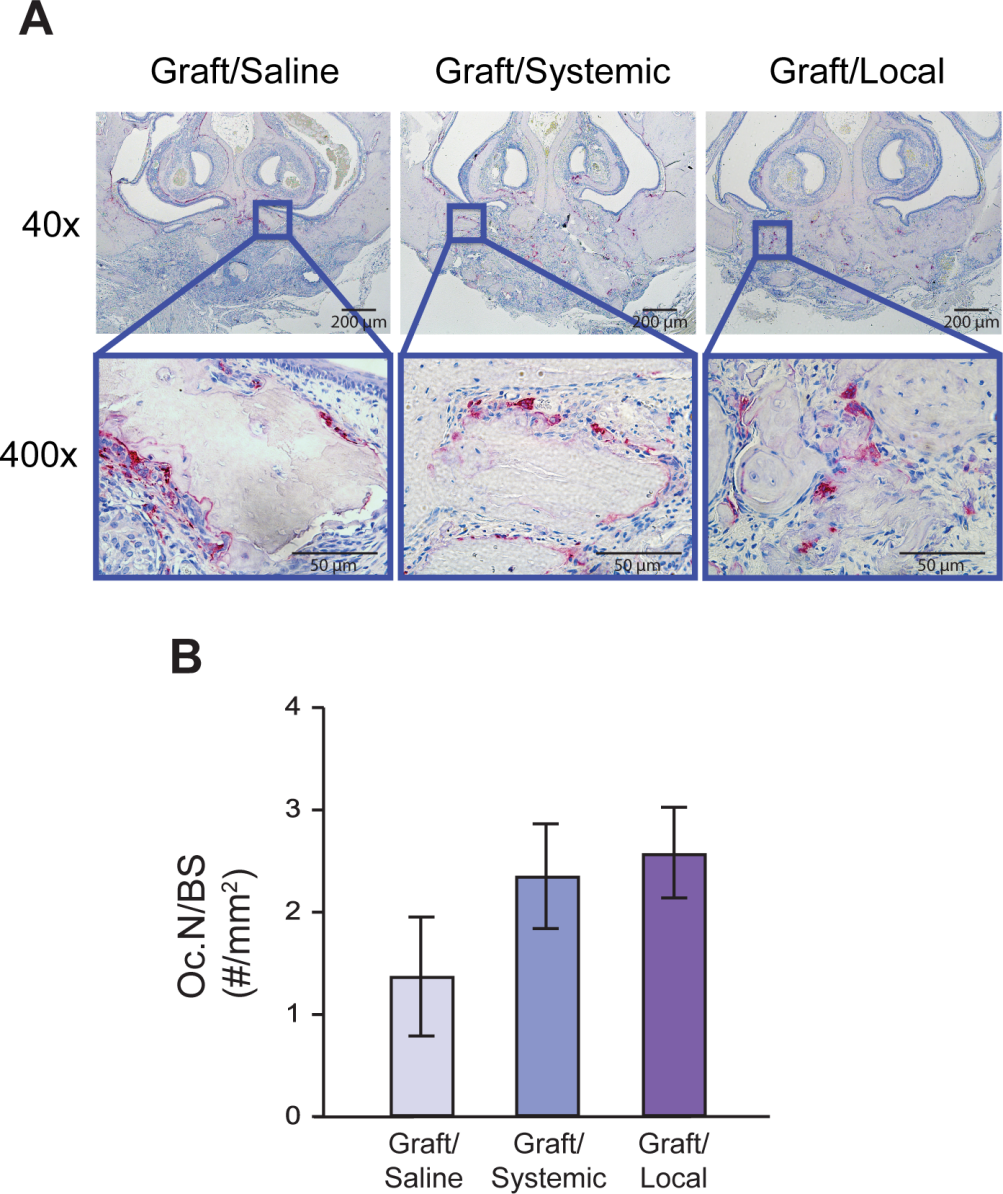
TRAP staining images and quantification. (A) TRAP staining at 40x and 400x magnification for all three groups. (B) Measurement of the number of TRAP+ cells per bone surface (Oc.N/BS) for each group.

**Fig 5:**
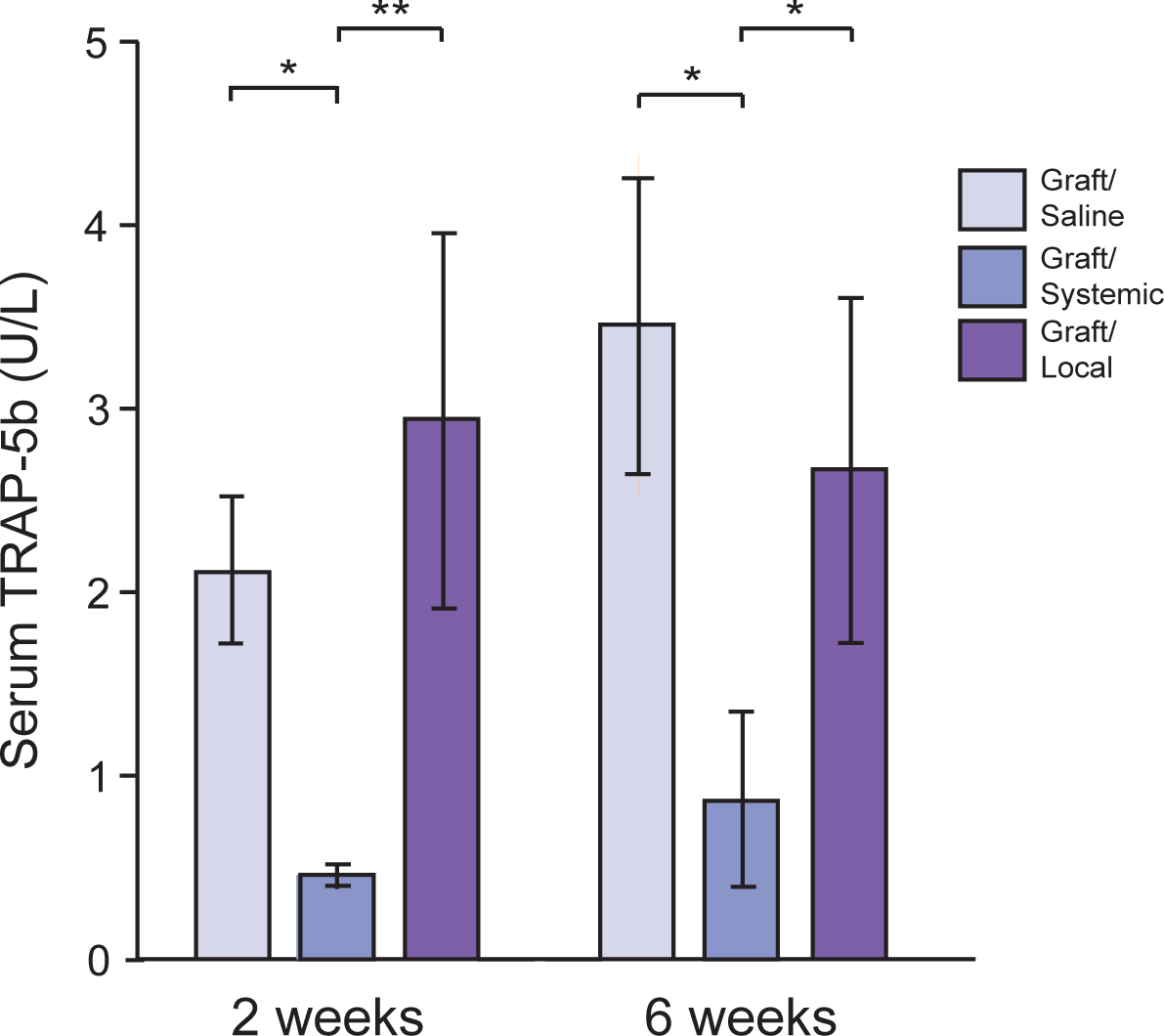
TRAP-5b levels using ELISA assay. Serum TRAP-5b levels analyzed by ELISA assay in the three experimental rat groups at 2 weeks and 6 weeks. * = p < 0.05, ** = p < 0.01.

To evaluate BP’s anti-osteoclastic effects, TRAP staining was performed (Fig 4A) and serum TRAP-5b levels were analyzed (Fig 5). An atypical morphology of round, detached TRAP+ cells was noted in both BP-treated groups (Fig 4A). TRAP staining showed no significant differences in quantity of osteoclasts per bone surface (Oc.N/BS) among the three groups (Fig 4B). TRAP-5b levels were significantly suppressed up to week 6 in the Graft/Systemic group (week 2: 0.46±0.07, week 6: 0.86±0.49U/L), compared to both the Graft/Local and Graft/Saline groups. By contrast, the Graft/Local group (week 2: 2.94±1.01, week 6: 2.67±0.99U/L) had minimal effects on peripheral osteoclast function, as its serum TRAP-5b levels were not significantly different from that of the Graft/Saline group (week 2: 2.11±0.47, week 6: 3.46±0.97U/L) (Fig 5).

## Discussion

The treatment of orafacial cleft patients requires comprehensive sequential treatment, including maxillary expansion followed by a bone graft surgery [9]. However, clinicians treating cleft patients have long struggled with high bone graft failure rates, compromising functionality, subsequent treatment, and esthetic outcomes, requiring additional surgeries. Graft incorporation failure is mainly caused by an imbalance of anabolic and catabolic activity during bone healing. As such, anabolic agents including TGF-beta and bone morphogenic proteins (BMPs) were previously utilized; however, the results demonstrated mixed outcomes [17,18]. Therefore, in this study, we used anti-resorptive agents, BPs, to target the excessive bone resorption during bone grafting and showed that BPs can effectively improve bone grafting outcomes. Furthermore, we also compared the effectiveness of systemic and local delivery of BPs in enhancing bone graft success.

While genetically engineered orofacial cleft rat models exist, these animals do not survive postnatally, requiring defect creation to study cleft bone grafting. Calvarial CSDs are well established; however, they do not incorporate important features of the oral environment including the presence of saliva, bacteria, and anatomical features such as immediate contact between soft and hard tissues [19]. Therefore, we developed an easily reproducible, intraoral CSD/bone graft model in rats (Fig 2A) to evaluate the bone graft procedure in the oral setting as in craniofacial patients. Most cleft patients undergo alveolar bone graft as a part of cleft repair and to aid tooth eruption in the cleft region. However, our CSD/bone graft is generated in the palate and may not accurately represent alveolar bone grafting.

In our previous study, it was determined that a delayed systemic injection of BP enhanced bone regeneration, while immediate BP administration at the time of surgery negatively affected bone graft incorporation as demonstrated by significantly lower BV/TV and MA/TA in Graft/BP/T0 group compared to Graft/saline and Graft/BP/T1 groups [16]. In order to determine the optimal method of delivery, the Graft/Local group was compared with the Graft/Systemic group, which received delayed BP injection one-week post-operatively.

Our study showed that BPs enhanced bone graft success as demonstrated by increased bone volume through microCT and histological analyses (Figs 2 and 3). Indeed, the observation of increased bone retention in BP-treated groups compared to control is consistent with the known inhibitory action of BPs on osteoclast function. Interestingly, the number of osteoclasts observed in histological analysis did not decline in BP-treated groups (Fig 4B). Instead, there was no statistical difference in osteoclast quantity. Our results support the findings of previous studies including Kaynak et al., who showed that only the morphology of osteoclasts diverged statistically between BP-treated and control groups, not their quantity [20–23]. In our study, an atypical morphology of round, detached TRAP+ cells was also noted in both BP-treated groups, indicative of disrupted osteoclast ultrastructure and function (Fig 4A).

To evaluate the osteoclastic activity as a function of time, serum TRAP-5b levels were assayed through ELISA. TRAP is highly expressed by mature osteoclasts. In serum, two distinct isoforms exist with TRAP-5b being specific to osteoclasts [24]. Therefore, it is a sensitive measure of osteoclastic activity. Surprisingly, substantially lower levels of TRAP-5b were maintained in the Graft/Systemic group up to week 6, compared to the Graft/Saline group, denoting that a single subcutaneous injection of BP was able to affect osteoclasts throughout the body for an extended period of time. Levels of TRAP-5b did not differ statistically between the Graft/Saline and Graft/Local groups, suggesting that by pretreating the graft with BPs through soaking, its action is confined to the defect area with minimal systemic influence, thereby decreasing the potential for adverse effects of BPs.

Interestingly, histological examination revealed increased new bone formation and bone incorporation, suggesting that BPs may have pro-osteogenic activity. Some previous studies attributed the increase in bone mass to a relative increase in bone formation rates due to a decrease in resorption rates without stimulation of osteoblastic activity [21,25]. However, our histological findings revealed osteoblast and osteocyte proliferation in the BP-treated groups, confirming recent studies which demonstrated BPs’ additional function in stimulating differentiation, maturation, and proliferation of osteoblasts *in vitro* [26–28]. Similarly, Altundal et al. found that systemic, repeated alendronate injections following autogenous grafting in rats resulted in increased biomarkers for bone formation, as well as a greater quantity of osteoblasts and osteoid formation [29]. Although the role of BPs in activating anabolic bone formation has been proposed, its mechanism of action remains unclear.

Our study suggests that the minimal systemic effects and ease of procedure associated with local administration of BPs confers the greatest enhancement in cleft bone grafting outcomes. Specifically, bone grafts were soaked in a low dose BP solution (0.005 mg/ml) for a short duration (3 min), followed by thorough rinsing to remove excess BPs to minimize any potential toxic effects of BPs as unbound BPs in the graft were previously shown to impair new bone formation [30]. While we followed the previously established approaches [10] in this study, this methodology does not control for the concentration of BP binding to bone graft materials and effective dose of BP. Jakobsen et al. investigated a dose-response effect of BPs using the most potent BP compound, zoledronic acid, and found that the lowest dose (0.005 mg/ml) increased new bone formation and decreased inhibition of allograft resorption while the inverse was seen in the highest dose (0.5 mg/ml) group [10]. Therefore, the affinity of BPs to the bone, concentration of the BP solution, and soaking time may be critical factors [31]. Further studies to include different classes of BPs and varying BP concentrations and immersion duration would aid in defining the optimal conditions for local delivery of BPs for future clinical applications.

Despite their favorable therapeutic effects, BPs must be used with caution to avoid potential adverse effects. Of greatest concern to dental professionals is the risk of developing BRONJ. However, osteoporotic patients with BP administration at low dose and frequency revealed a rare incidence of BRONJ [32]. In addition, the risk of BRONJ in children is further reduced due to high bone turnover; indeed, pediatric BRONJ has yet to be reported [33]. Other dental concerns include tooth eruption and orthodontic tooth movement. Indeed, BPs are known to delay tooth eruption [34,35] and while orthodontic treatment is not contraindicated in patients undergoing BP treatment, complications such as delayed tooth movement, incomplete space closure, and longer treatment duration have been observed [36]. However, complete inhibition of tooth eruption and movement is rare, possibly due to high bone turnover and short BP retention in children. While inhibition of tooth eruption and movement to achieve maxillary alignment in cleft patients is not anticipated at the low clinical dose of 0.1mg/kg administered in this study, use of another class of BPs with low potency such as first generation BPs might be a potential alternative to zoledronic acid to facilitate tooth eruption to the grafted area.

Further studies are required to evaluate the dental concerns associated with BP use as they may play an important role in determining treatment plans in comprehensive craniofacial patient care. In addition, as orofacial cleft repair requires both hard and soft tissue repair, examination for enhancement of soft tissue regeneration is required as this approach translates into more precise standardized protocol. The current study is further limited as our murine model does not completely simulate the orofacial cleft repair in human. Murine bone exhibits different characteristics than human bone, especially with respect to the craniofacial complex. Hence, the results from this model are important preclinical data, but not immediately applicable to clinical situations.

## Supporting information

S1 Table Raw data for main figures. (A) BV/TV from Fig 2B (B) BMD from Fig 2C (C) MA/TA from Fig 3B (D) BG/TA from Fig 3C (E) Oc.N/BS from Fig 4B (F) Serum TRAP-5b at 2 week and 6 week time points from Fig 5.
(DOCX)
