# Expression of Concern: Local vs. systemic administration of bisphosphonates in rat cleft bone graft: A comparative study

**DOI:** 10.1371/journal.pone.0344844

**Published:** 2026-03-13

**Authors:** The PLOS One Editors

After this article [1] was published, concerns were raised regarding results presented in Figs 2-4.

Specifically:

- The Fig 2A Control panel of this article [1] appears similar to the Fig 4a Control panel in [2], despite the articles reporting different ages of the rats included in these control groups.
- The Fig 3A Control 40X and 100X images of this article [1] appear similar to the Fig 5a Control 4X and 10X images in [2], despite the articles reporting different ages of the rats in these control groups.
- The number of animals in each group in Fig 1A and the S1 Table appears different.
- The underlying data were not provided in the published article [1] contrary to the Data Availability statement.

Regarding Figs 2A and 3A in [1], the corresponding author stated that [1] and [2] were part of a larger project to evaluate the effect of bisphosphonates on cleft bone grafting, with [1] focusing on the method of delivery and [2] focusing on the timing of treatment delivery. They stated that the Control group was performed only once, simultaneously with the experiments in [2], and was not repeated for the experiments in [1]. The Control group images in Figs 2A and 3A in [1] were included as a visual demonstration only of the novel animal model developed in the project, and the Control group was not included in the statistical analyses of these figures in [1].

The corresponding author stated that in [1], the reported age of 20 weeks for the rats is incorrect and the correct age is 15 weeks.

A member of the *PLOS One* Editorial Board reviewed Fig 2A and 3A in [1] and the corresponding author’s response. They noted that Fig 2C in [1] and Fig 4C in [2] appear to show different results for the Control group, and that the Fig 3A Control images in [1] are labeled as 40X and 100X, whereas the Fig 5a Control group images in [2] are labeled as 4X and 10X. The corresponding author states that the data in Fig 2C in [1] are correct and the wrong units were reported in Fig 4C in [2]. They state that the magnification reported for Fig 3A in [1] is incorrect and should instead read 4X and 10X. They provided an updated version of Fig 3 with the correct magnification, and updated versions of Figs 2-4 where the “Control group” label is updated to “Negative Defect”. With these explanations and updated figures, PLOS considers the concerns pertaining to the Control/Negative Defect group results in Figs 2C and 4C in [1], as well as the magnification of the images in Figs 2A and 3A, resolved.

The corresponding author provided the data underlying the results in [1] (S1-S4 Files). Upon editorial review, it was noted that there appear to be differences between the underlying quantitative data in S1 File and S1 Table in [1]. Specifically:

- In the following results, the standard deviation in S1 File appears different to the error bars in the published figures in [1]: Figs 2C, 3B, 3C, 4B.
- The following results appear different between S1 Table in [1] and S1 File:
  - Fig 2C BMD Control mean and standard deviation
  - Fig 3B Graft/Saline mean and the number of animals (N) in all conditions
  - Fig 4B standard deviations

The corresponding author stated that errors occurred in the preparation of Figs 2-5, and they provided updated versions of Figs 2-5 which are provided here. Regarding the differences between S1 Table in [1] and S1 File provided with this notice, the corresponding author stated that in S1 Table in [1], the following values are incorrect:

A: BV/TV (%) Control number of animals (N)
B: BMD (g/cm^3^) Control Mean, SD, and number of animals
C: MA/TA (%) number of animals (N) for all conditions
D: BG/TA (%) Graft/Local SD
E: Oc.N/BS (#/mm^2^) SD for all conditions

Upon editorial review, PLOS noted that multiple means and standard deviations reported in the Results section in [1] are incorrect, and readers should refer to S1 File provided with this notice for the correct values. The corresponding author stated the statistical significance of the results are not affected by these issues, and provided the underlying GraphPad Prism files for the updated Figs 2-5 in S5 File. The *PLOS One* Editors note that compared to Fig 5 in [1] which shows p < 0.05 (*) for the 6 weeks Graft/Saline comparison with 6 weeks Graft/Systemic, the updated Fig 5 for this comparison shows p < 0.01 (**). Based on S1 File, the following values in the Results section of [1] are incorrect:

- In the first sentence of the third paragraph of the Effect of BP on bone grafting subsection, “Graft/Saline group (19.74 ± 18.89%)” should read “Graft/Saline group (19.75 ± 18.89%)”;
- In the fourth sentence of the third paragraph of the Effect of BP on bone grafting subsection, “Graft/Local (16.95 ± 3.41%)” should read “Graft/Local (16.95 ± 3.46%)”;
- In the fourth sentence of the first paragraph of the Effect of bisphosphonate on osteoclast activity subsection, “Graft/Systemic group (week 2: 0.46 ± 0.07, week 6: 0.86 ± 0.49U/L)” should read “Graft/Systemic group (week 2: 0.47 ± 0.07, week 6: 0.86 ± 0.49U/L)”.

Additionally, the corresponding author provided an updated Fig 1 (S6 File) where the Control group label is changed to Negative Defect. The *PLOS One* Editors note that the Negative Defect group in S6 File and Control group in the published Fig 1A [1] show n = 4; however, S1 File shows the number of animals in the Control group for Figs 2B and 2C as 3. The corresponding author states that 4 rats were assigned to the Control group in [1], with 3 included in the final analysis, and 10 rats were assigned to the Graft/Saline, Graft/Systemic, and Graft/Local groups with 4–8 rats included in the final analysis. The *PLOS One* Editors noted that only 4 individual measurements were included in the S1 File data underlying Fig 3B. The corresponding author stated that for Fig 3B, the correct number of analyzed specimens (n number) is 4, and that the correct n number for Fig 3C is 5. The *PLOS One* Editors note that Figs 3B and 3C appear to report quantifications based on the same underlying image data, including the images presented in Fig 3A. PLOS remains concerned for the difference in the number of analyzed samples between these figures.

In light of the extent of the data reporting errors listed above, PLOS remains concerned about the overall reliability of the data as published in [1]. The *PLOS One* Editors issue this Expression of Concern to notify readers of the above issues, to relay the information and data provided, and to inform readers to interpret the results with caution.

Fig 1A, the Fig 2A Control panel, and the Fig 3A Control panel in [1] report material adapted from or previously published in [2], published in 2017 by Sage, which are not offered under a CC BY license and are therefore excluded from this article’s [1] license. Please provide due attribution to the original publication when referring to this content.

**Fig 2:**
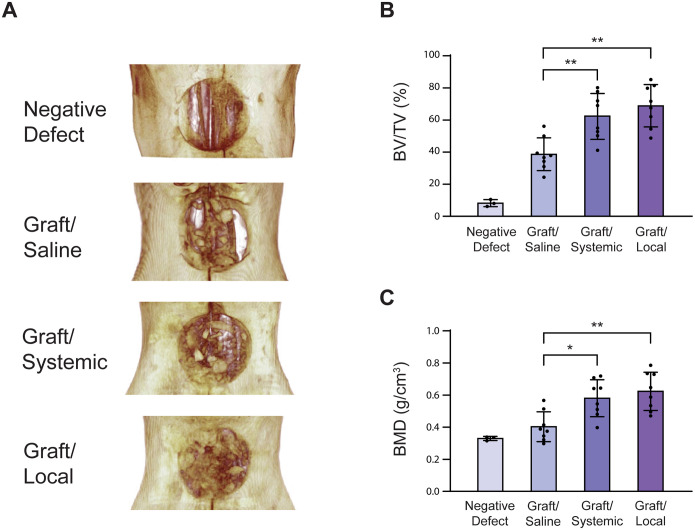
MicroCT images and 3D volumetric analysis. **(A)** 3D image reconstruction of palatal defect and grafting six weeks after surgery. Quantification of bone volume by volumetric analysis of microCT images for the experimental grafted groups: Graft/Saline (vehicle control), Graft/Systemic, and Graft/Local. **(B)** Bone volume fraction (BV/TV) (n = 8) and (C) bone mineral density (BMD) (n = 8). * = p < 0.05, ** = p < 0.01. The Fig 2A Negative Defect image was originally published in [2] and is excluded from this article’s CC BY 4.0 license. Please see the accompanying Expression of Concern for more information.

**Fig 3:**
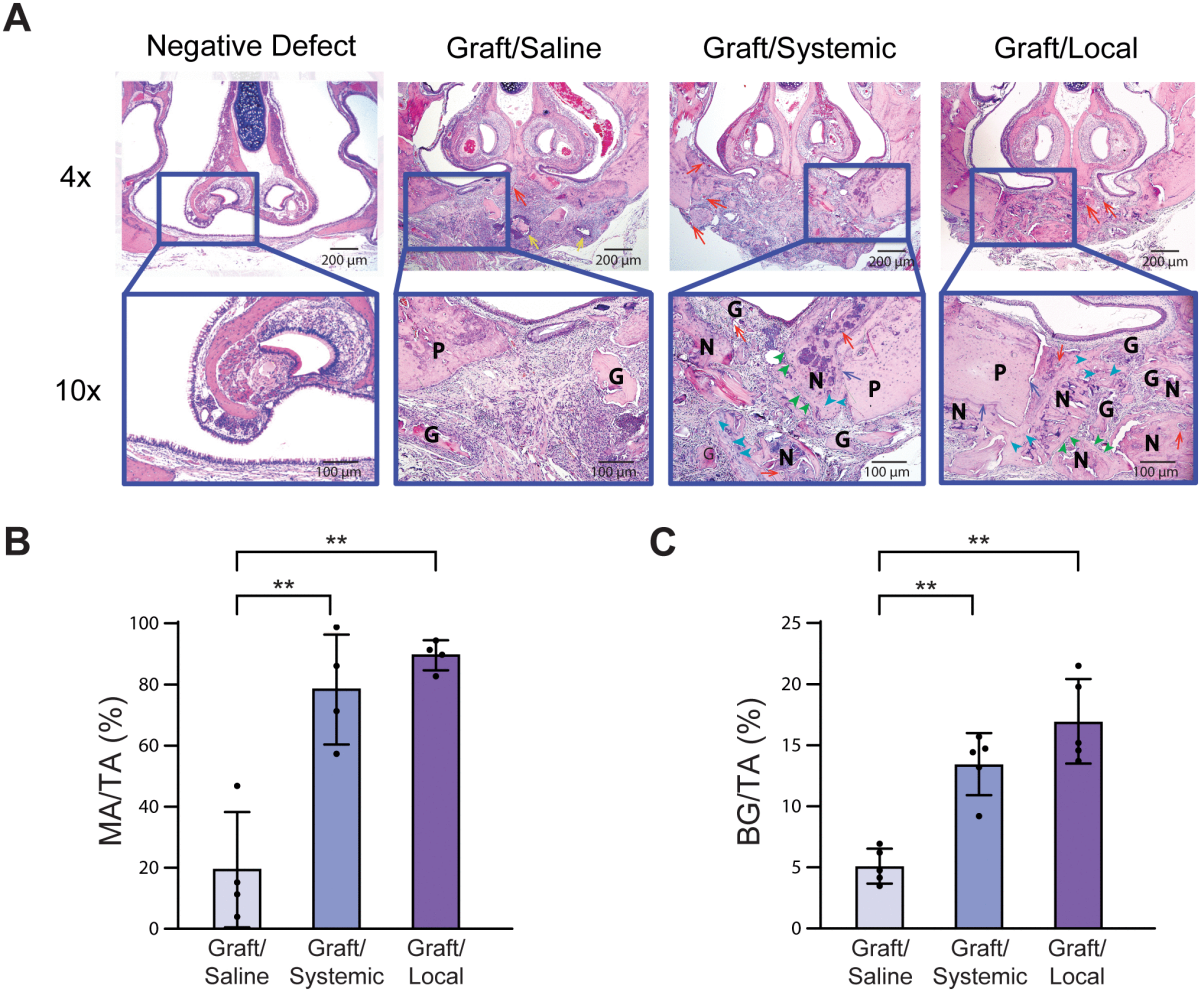
Histomorphometric analysis. **(A)** H&E-stained coronal sections at 4× and 10 × magnifications of all grafted experimental groups. G: graft; P: palatal bone; N: new bone; red arrows indicate blood vessels around bone graft particles; yellow arrows indicate acute inflammation; blue arrows indicate bone integration with defect margins; green arrowheads indicate osteoblasts; light blue arrowheads indicate osteocytes. **(B)** Quantification of mineralized area/total tissue area (MA/TA) using image analysis software (Advanced SPOT 4.6, Macomb County, MI) (n = 4). **(C)** Quantification of bone graft/total tissue area (BG/TA) using Advanced SPOT 4.6 software (n = 5). * = p < 0.05, ** = p < 0.01. The Fig 3A Negative Defect image was originally published in [2] and is excluded from this article’s CC BY 4.0 license. Please see the accompanying Expression of Concern for more information.

**Fig 4:**
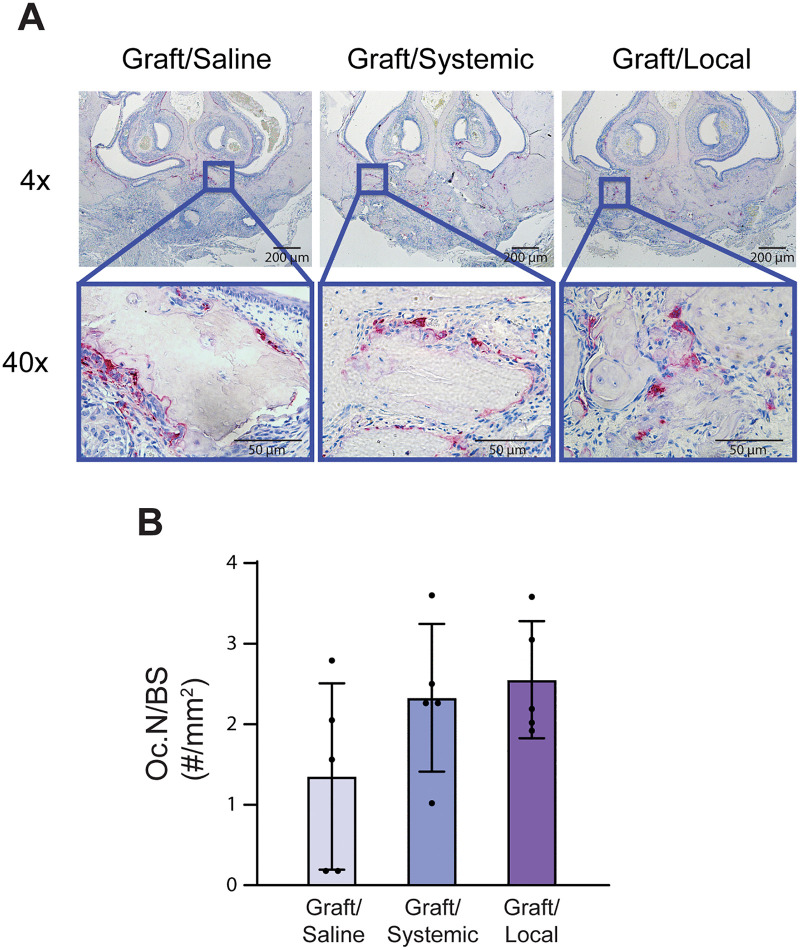
TRAP staining images and quantification. **(A)** TRAP staining at 4× and 40 × magnification for all grafted experimental groups. **(B)** Quantification of TRAP-positive cells per bone surface (Oc.N/BS) (n = 5).

**Fig 5:**
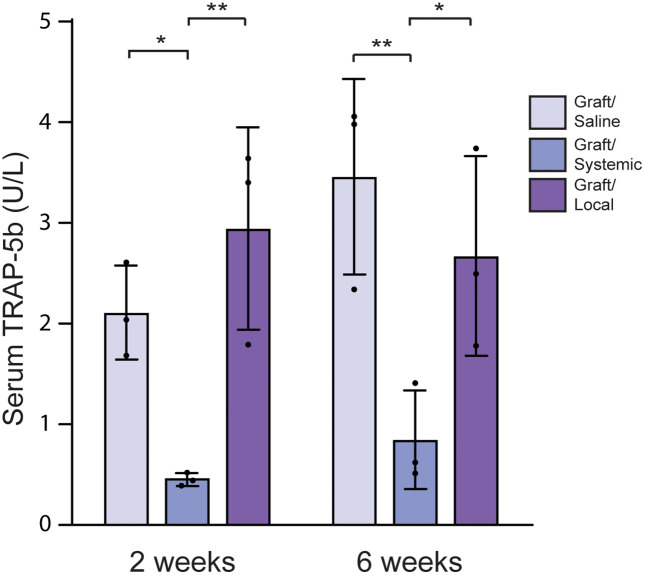
TRAP-5b levels using ELISA assay. Serum TRAP-5b levels analyzed by ELISA assay in the three experimental rat groups at 2 weeks and 6 weeks. * = p < 0.05, ** = p < 0.01.

## Supporting information

S1 File Underlying quantitative data for Figs 2–5. (XLSX)

S2 File Original image data underlying Fig 2A. (PDF)

S3 File Original image data underlying Fig 3A. (PDF)

S4 File Original image data underlying Fig 4A. (PDF)

S5 File Statistical analyses underlying Figs 2–5. (ZIP)

S6 File Alternative version of Fig 1. (TIF)
