# Supplementary material for: Local vs. systemic administration of bisphosphonates in rat cleft bone graft: A comparative study
Source: PLoS One. 2018 Jan 5;13(1):e0190901. doi: 10.1371/journal.pone.0190901 (PMC5755940; doi:10.1371/journal.pone.0190901)
Supplement: S1 Table — (A) BV/TV from Fig 2B (B) BMD from Fig 2C (C) MA/TA from Fig 3B (D) BG/TA from Fig 3C (E) Oc.N/BS from Fig 4B (F) Serum TRAP-5b at 2 week and 6 week time points from Fig 5. (DOCX) [file pone.0190901.s001.docx]

**Supporting Information**

| **A** |  |  |  |
| --- | --- | --- | --- |
| BV/TV (%) | Mean | SD^a^ | N^b^ |
| Control | 8.71 | 2.21 | 8 |
| Graft/Saline | 39.18 | 10.18 | 8 |
| Graft/Systemic | 62.99 | 14.31 | 8 |
| Graft/Local | 69.35 | 13.18 | 8 |

| **B** |  |  |  |
| --- | --- | --- | --- |
| BMD (g/cm^3^) | Mean | SD | N |
| Control | 0.31 | 0.101 | 8 |
| Graft/Saline | 0.41 | 0.09336 | 8 |
| Graft/Systemic | 0.59 | 0.115 | 8 |
| Graft/Local | 0.63 | 0.1192 | 8 |

| **C** |  |  |  |
| --- | --- | --- | --- |
| MA/TA (%) | Mean | SD | N |
| Control | 0 | 0 | 5 |
| Graft/Saline | 19.74 | 18.89 | 5 |
| Graft/Systemic | 78.76 | 18 | 5 |
| Graft/Local | 89.95 | 4.932 | 5 |

| **D** |  |  |  |
| --- | --- | --- | --- |
| BG/TA (%) | Mean | SD | N |
| Graft/Saline | 5.11 | 1.43 | 5 |
| Graft/Systemic | 13.45 | 2.54 | 5 |
| Graft/Local | 16.95 | 3.41 | 5 |

| **E** |  |  |  |
| --- | --- | --- | --- |
| Oc.N/BS (#/mm^2^) | Mean | SD | N |
| Graft/Saline | 1.352 | 0.517 | 5 |
| Graft/Systemic | 2.328 | 0.4103 | 5 |
| Graft/Local | 2.552 | 0.3254 | 5 |

| **F** |  |  |  |
| --- | --- | --- | --- |
| TRAP-5b (U/L) : 2 week | Mean | SD | N |
| Graft/Saline | 2.11 | 0.467 | 3 |
| Graft/Systemic | 0.466 | 0.065 | 3 |
| Graft/Local | 2.944 | 1.005 | 3 |
| 6 week |  |  |  |
| Graft/Saline | 3.459 | 0.971 | 3 |
| Graft/Systemic | 0.863 | 0.489 | 3 |
| Graft/Local | 2.672 | 0.992 | 3 |

^a^ : Standard deviation

^b^: Sample size
