# Supplementary figures and images for: Expression of Concern: Local vs. systemic administration of bisphosphonates in rat cleft bone graft: A comparative study
Source: PLoS One. 2026 Mar 13;21(3):e0344844. doi: 10.1371/journal.pone.0344844 (PMC12987411; doi:10.1371/journal.pone.0344844)

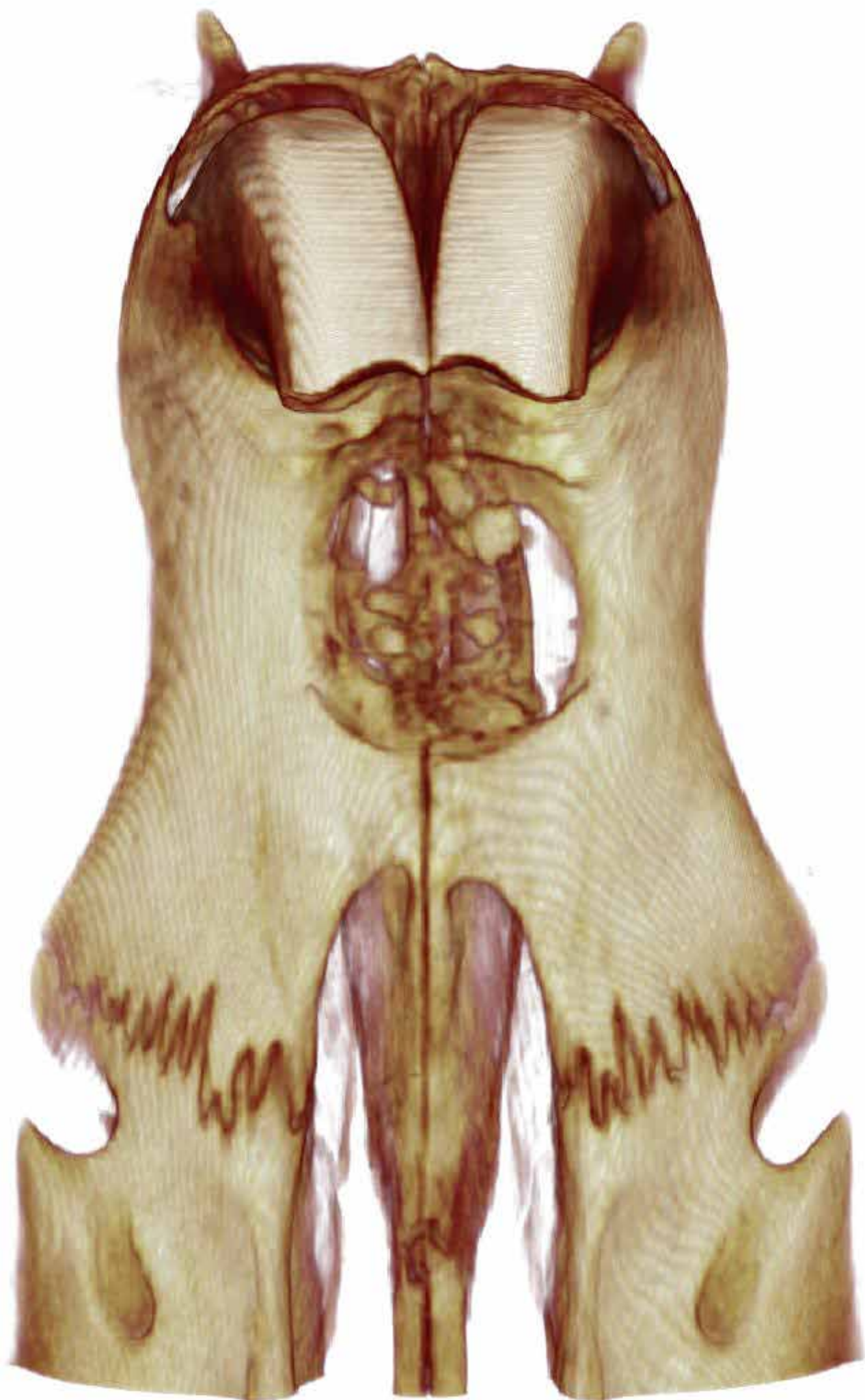

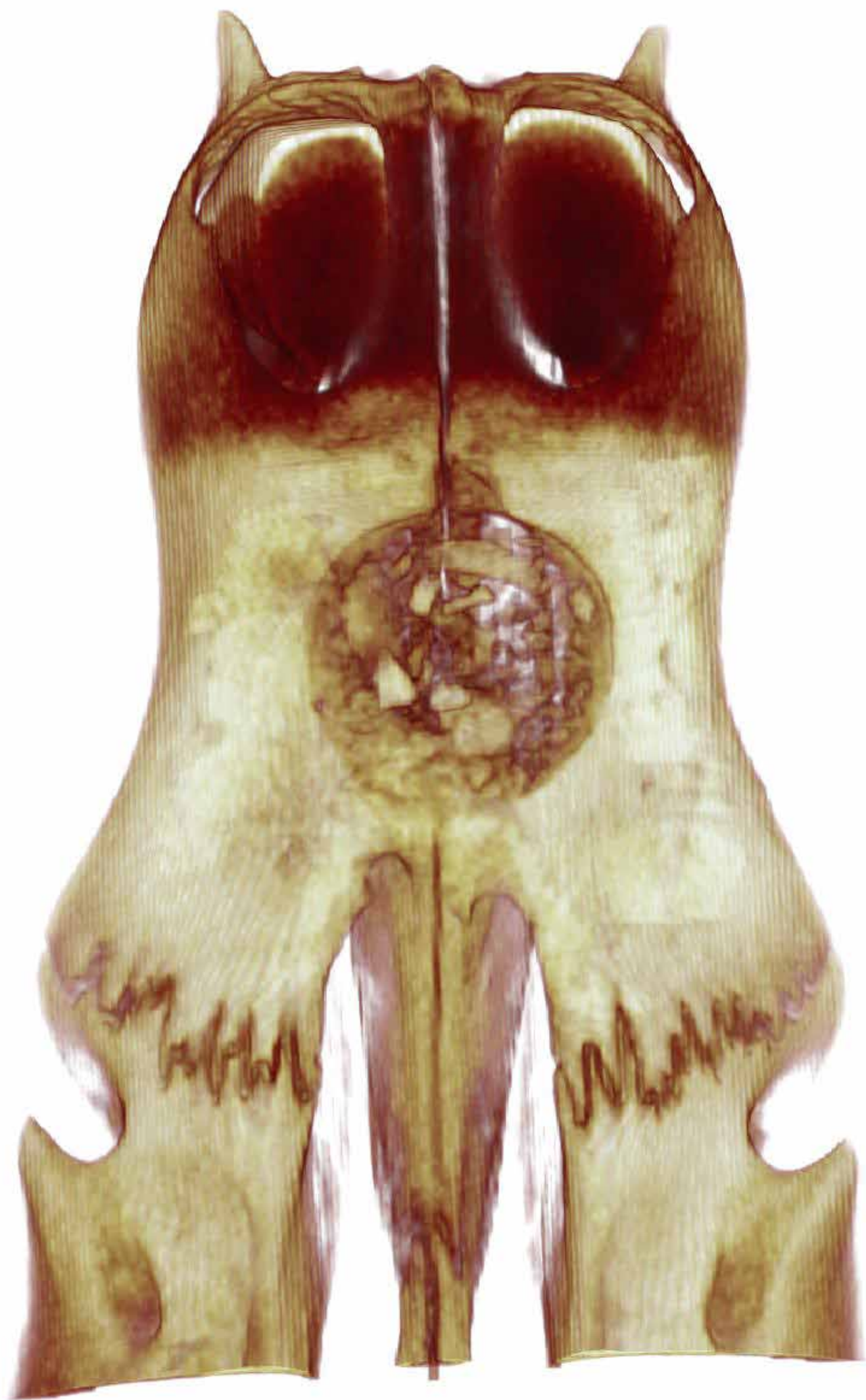

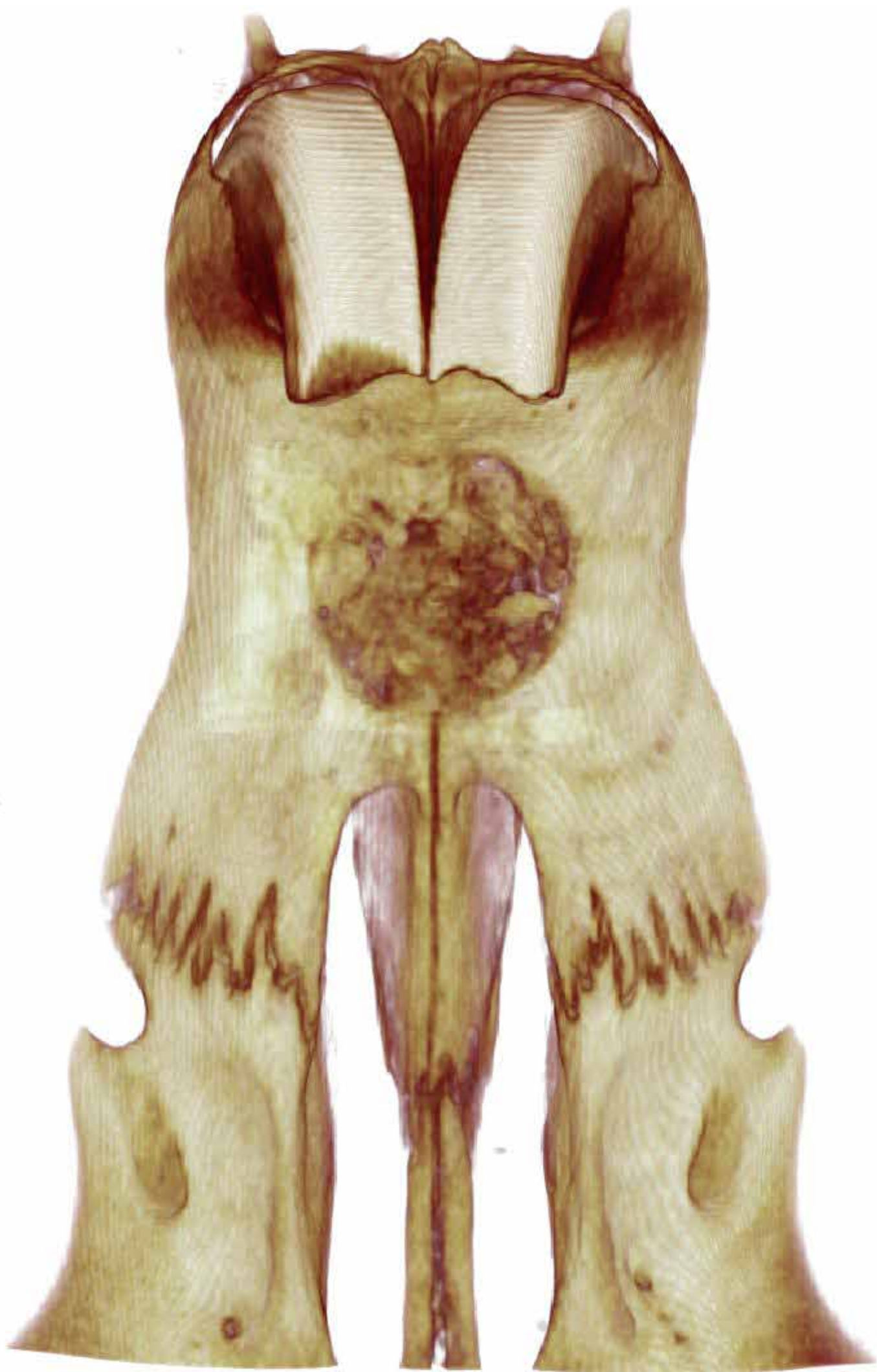

Supplement: S2 File — (PDF) [file pone.0344844.s002.pdf]

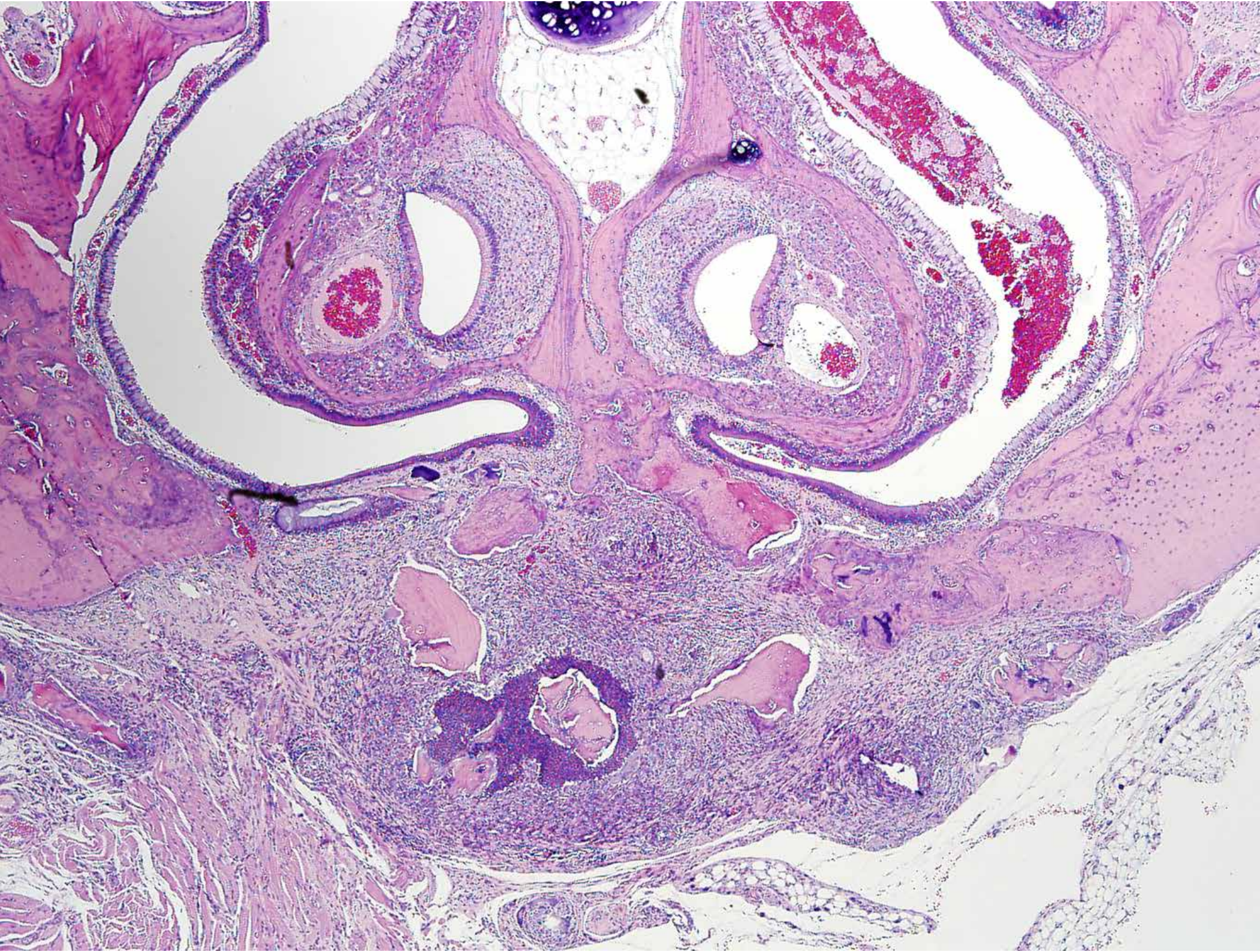

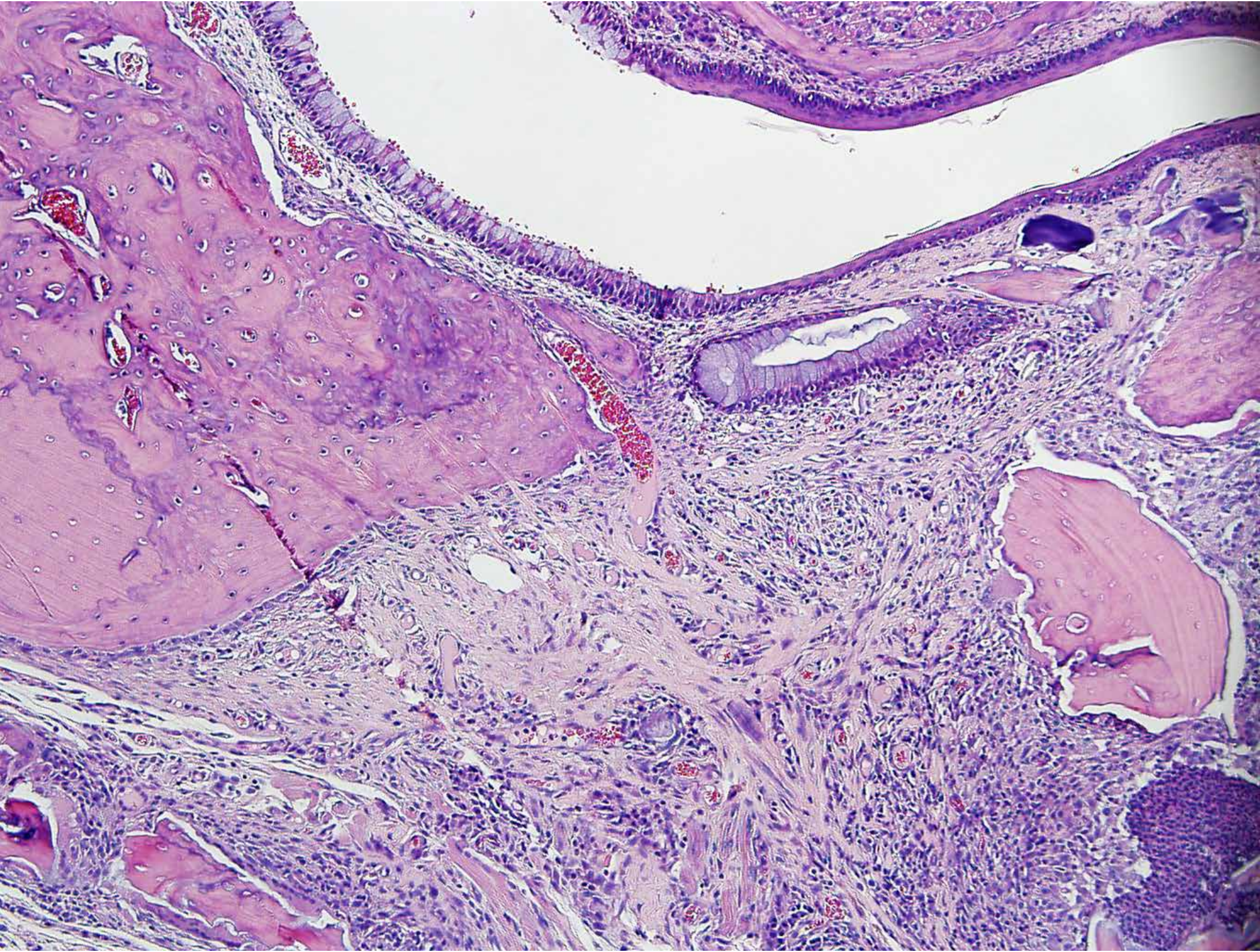

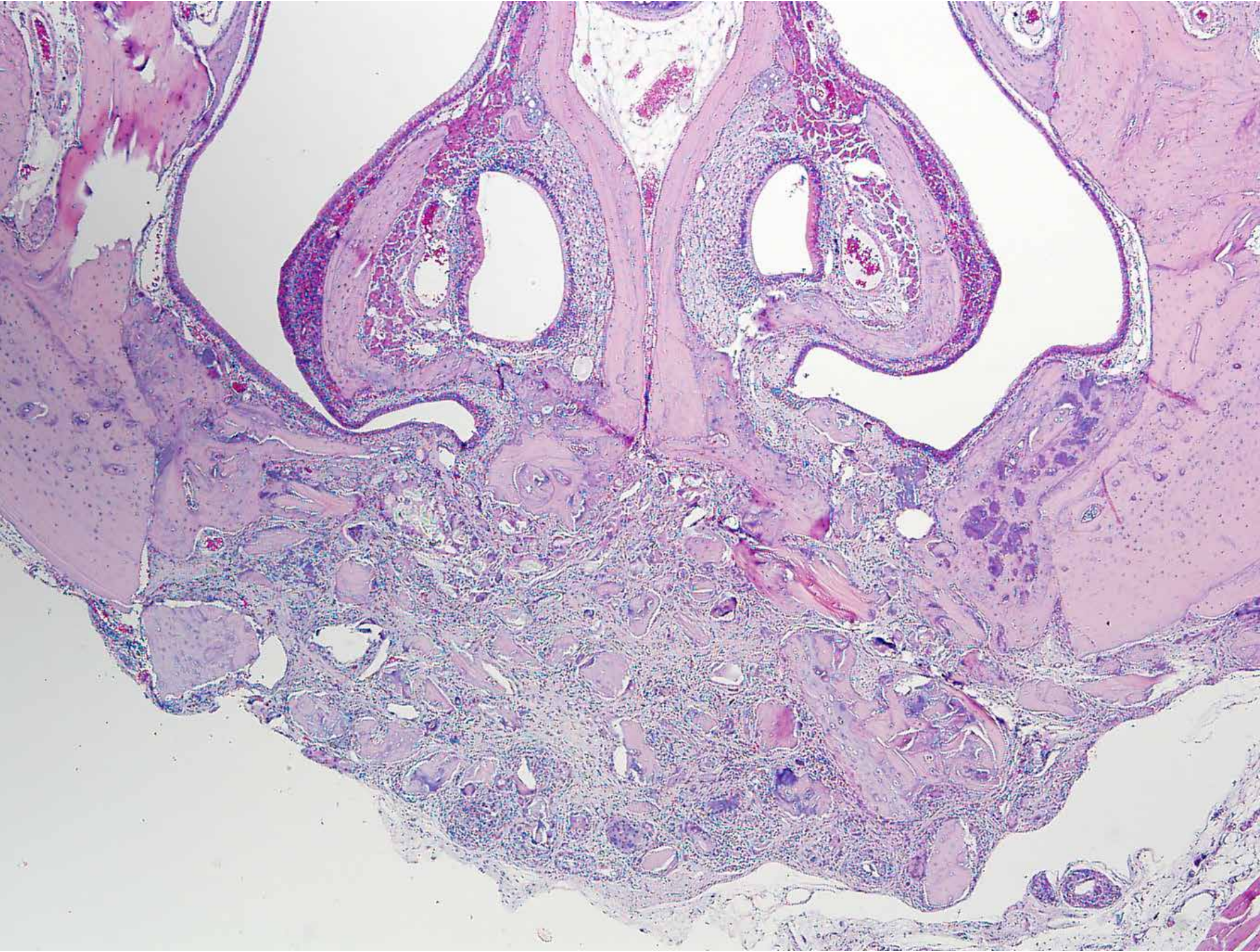

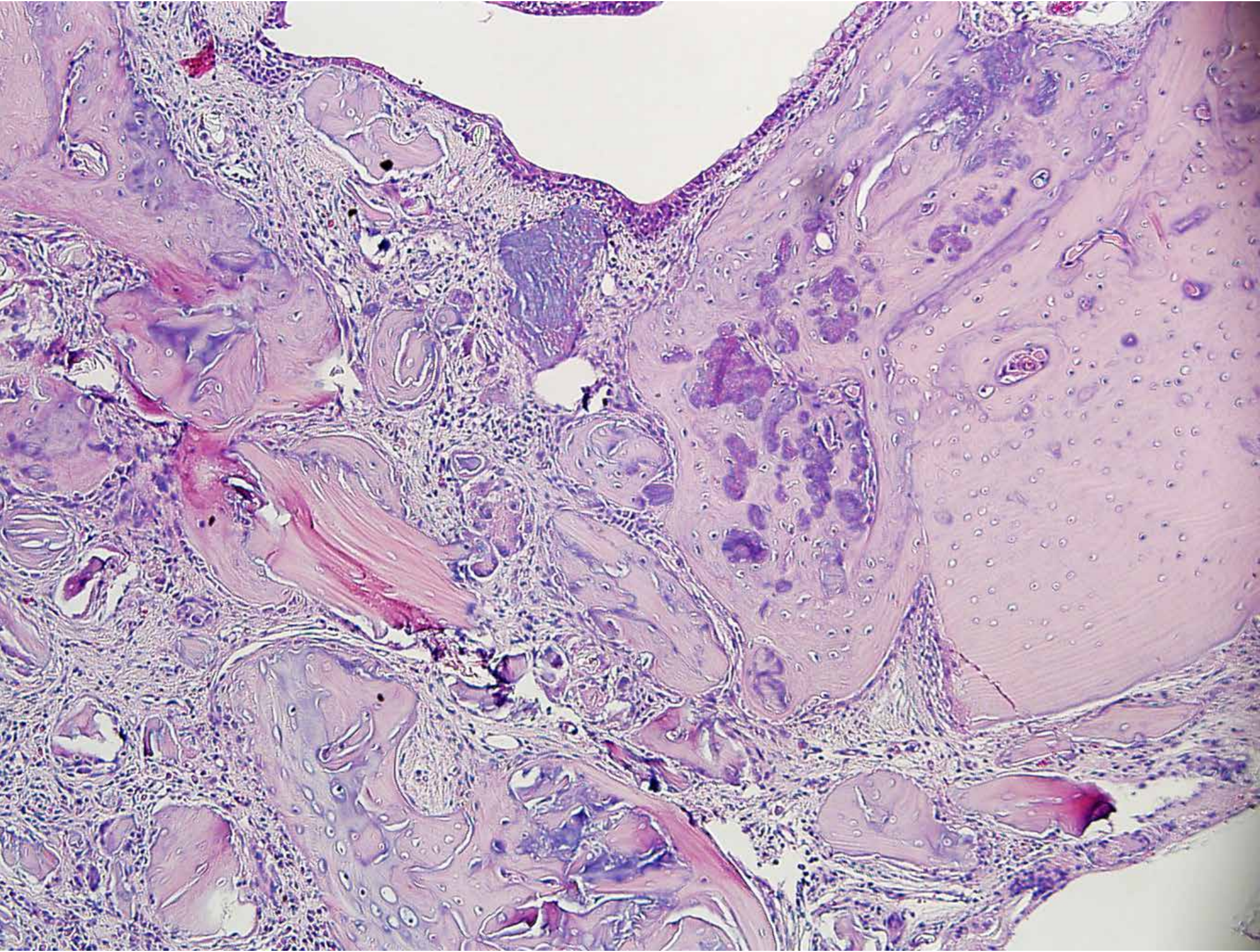

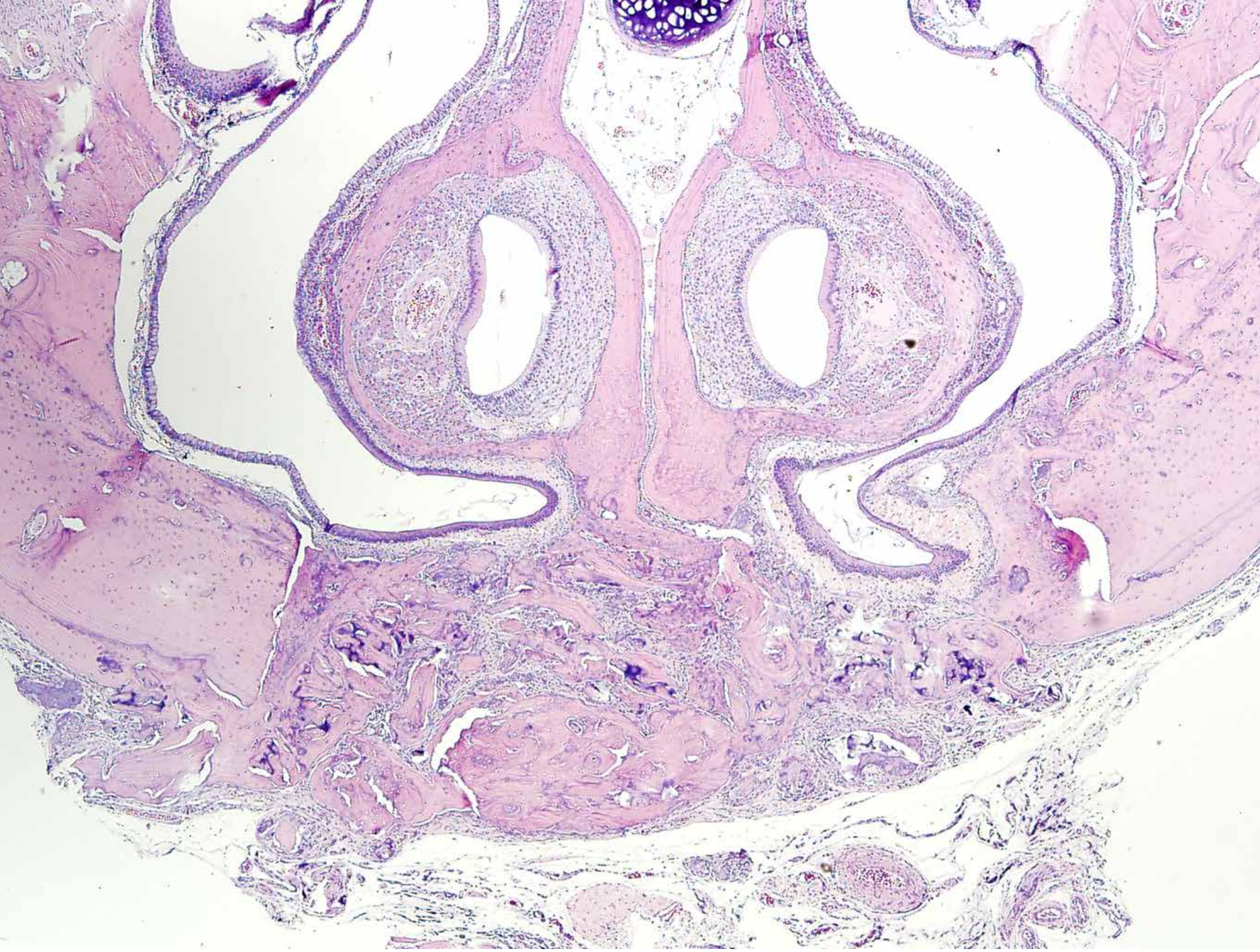

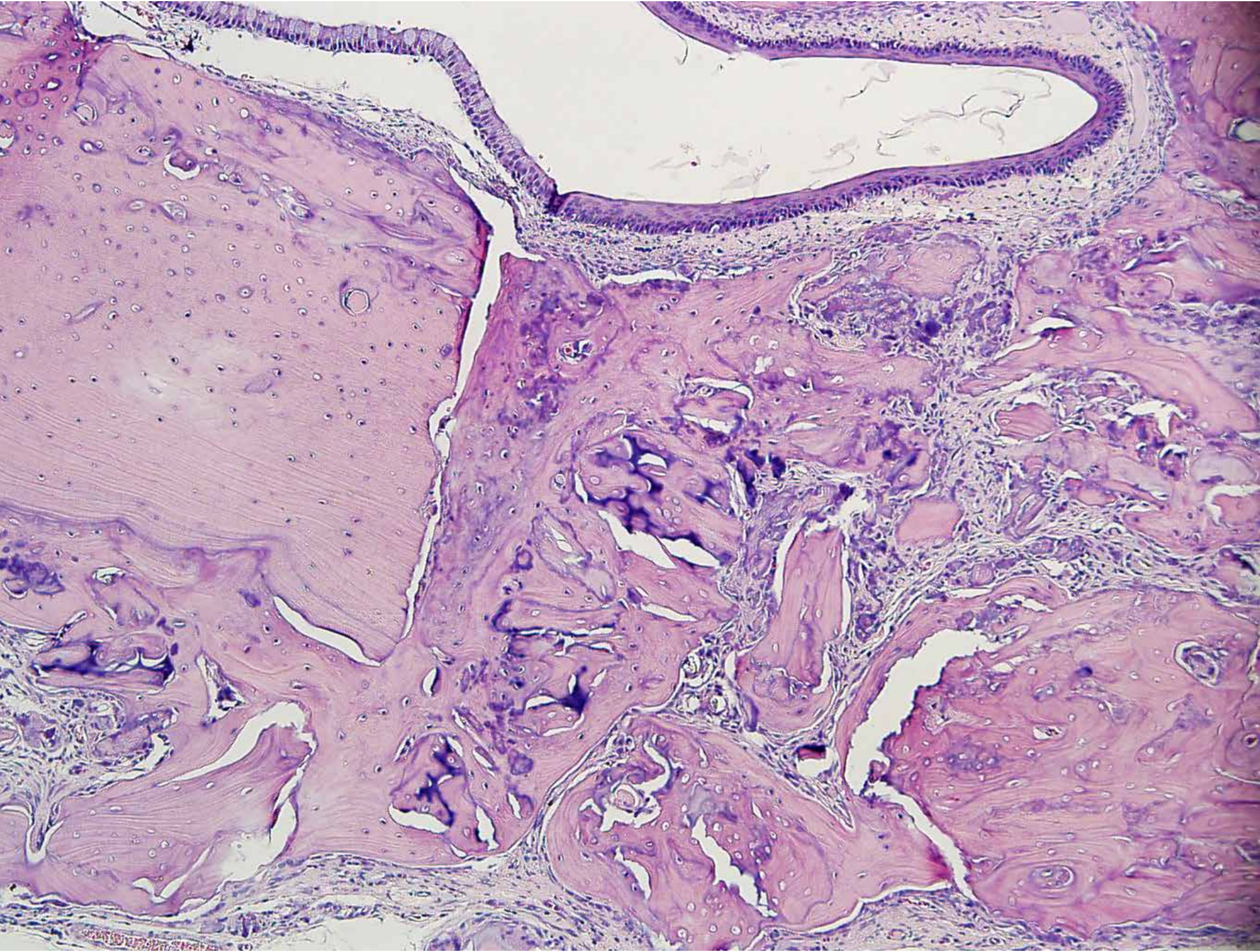

Supplement: S3 File — (PDF) [file pone.0344844.s003.pdf]

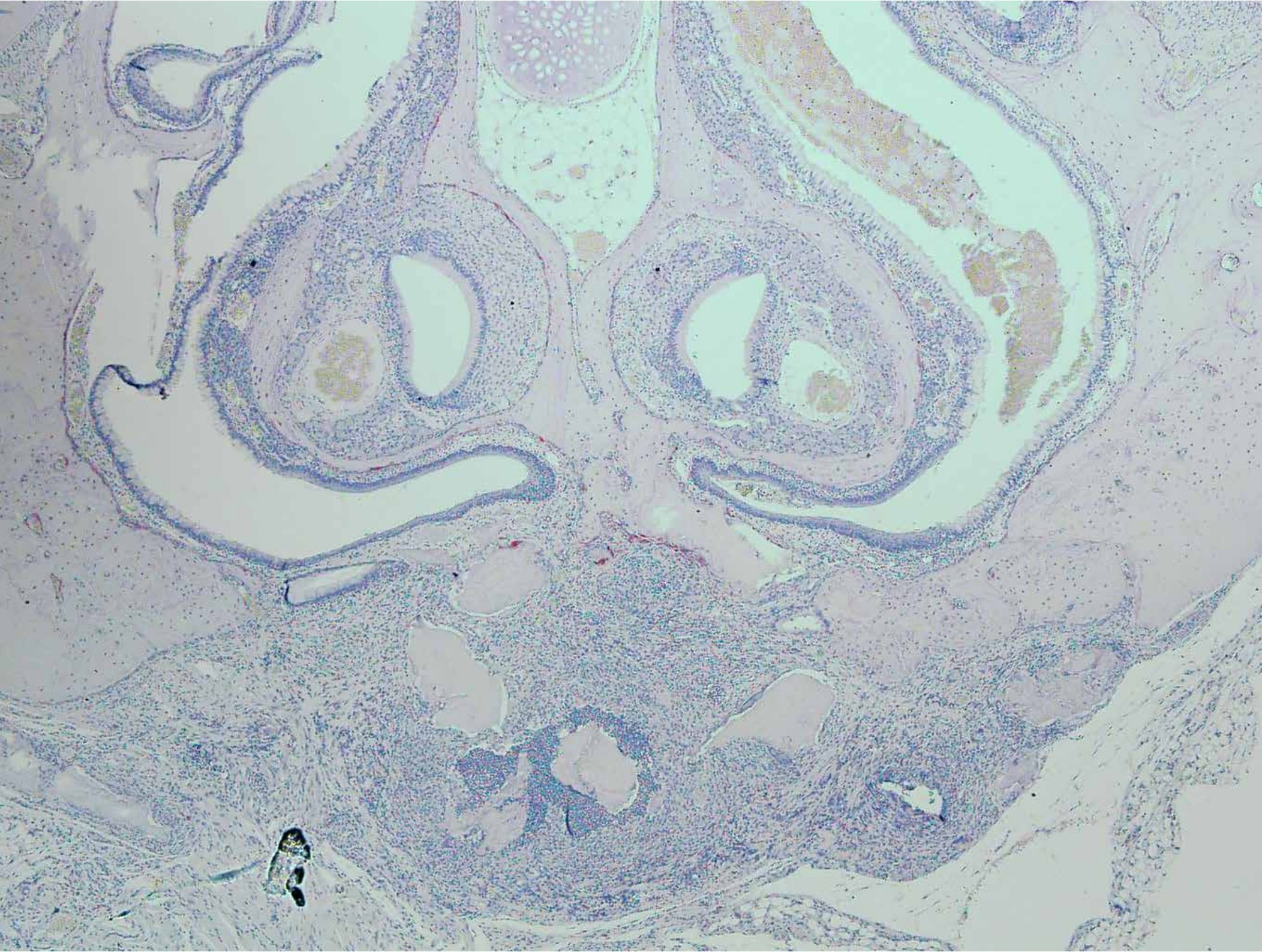

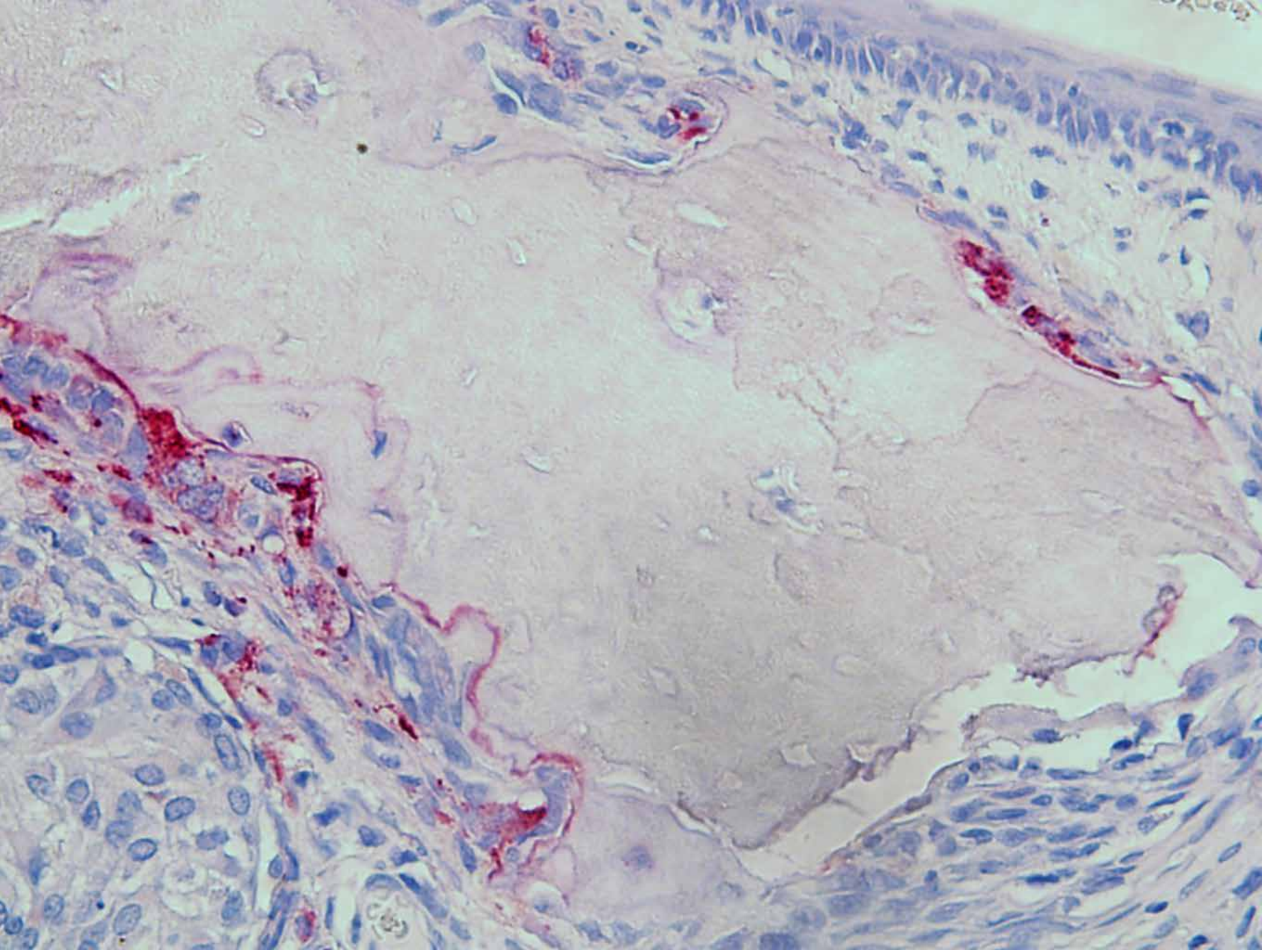

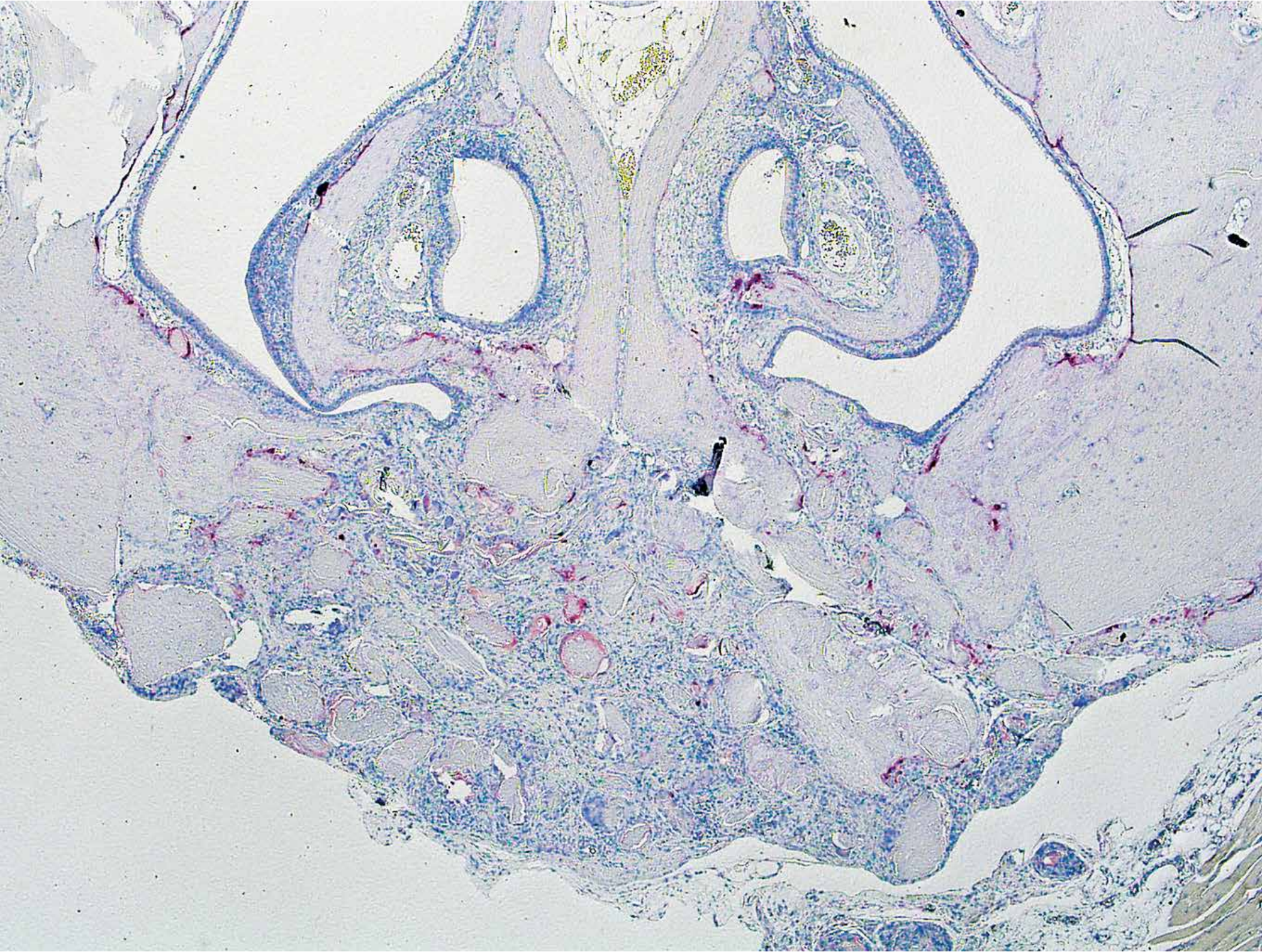

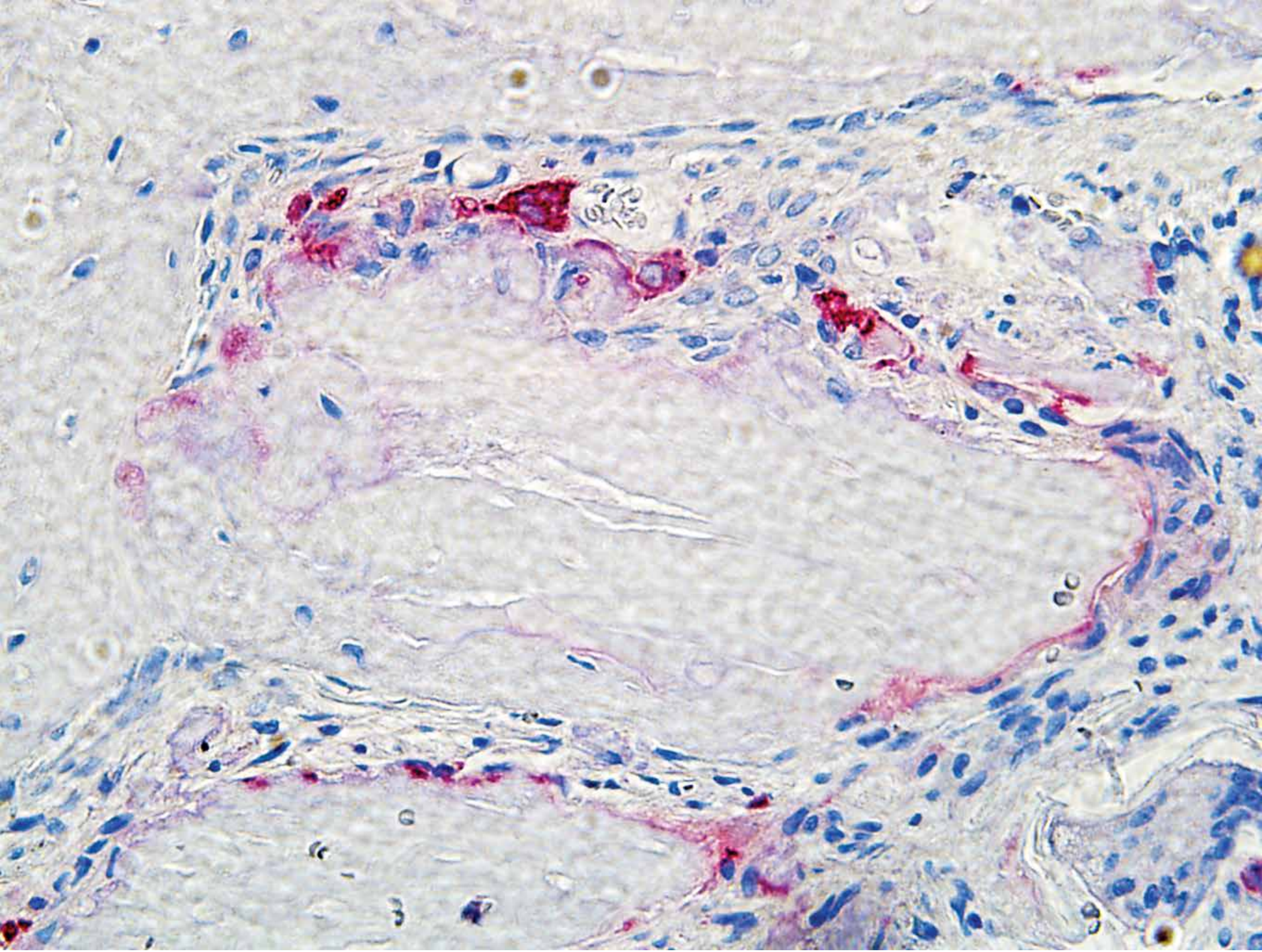

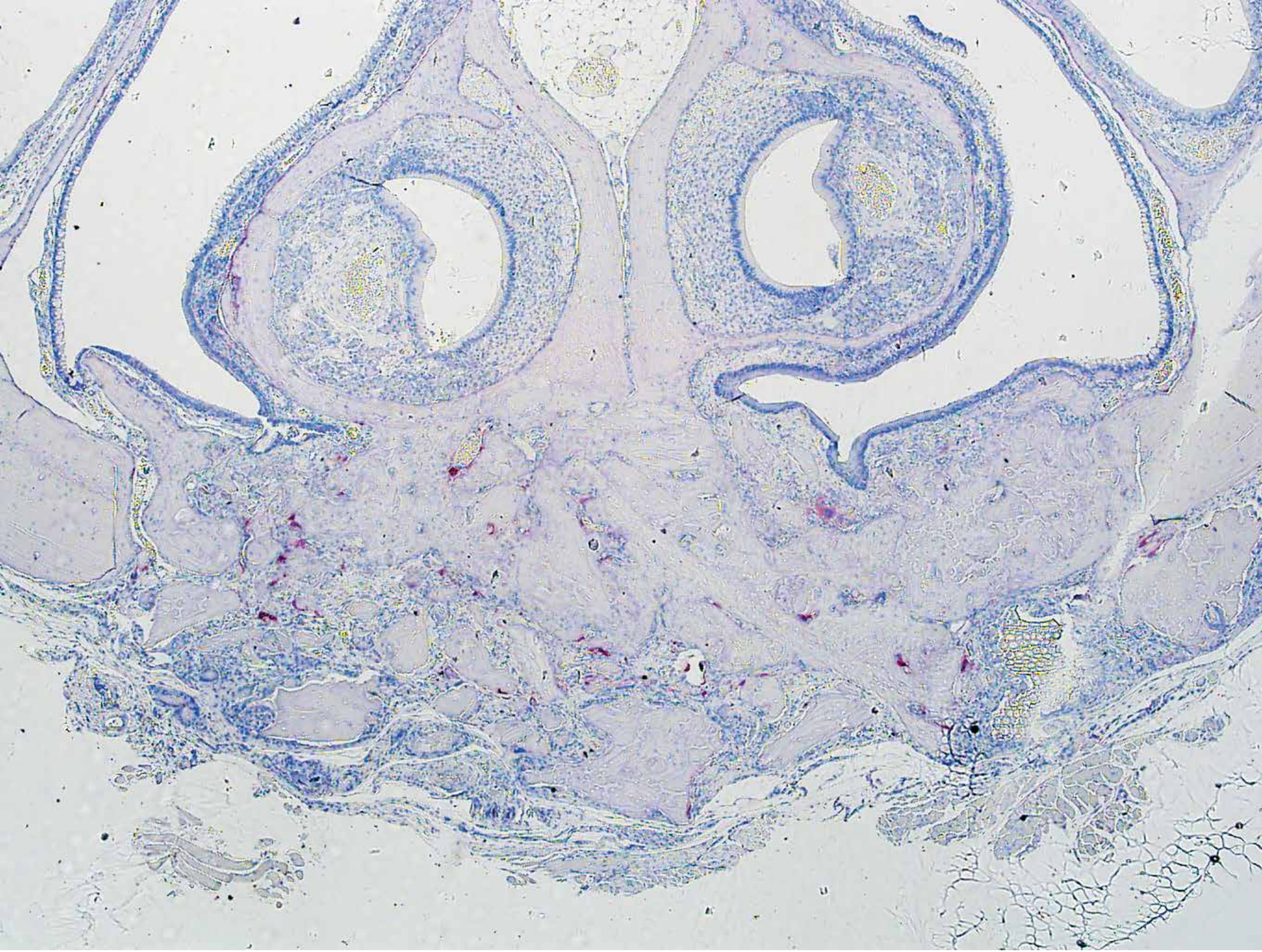

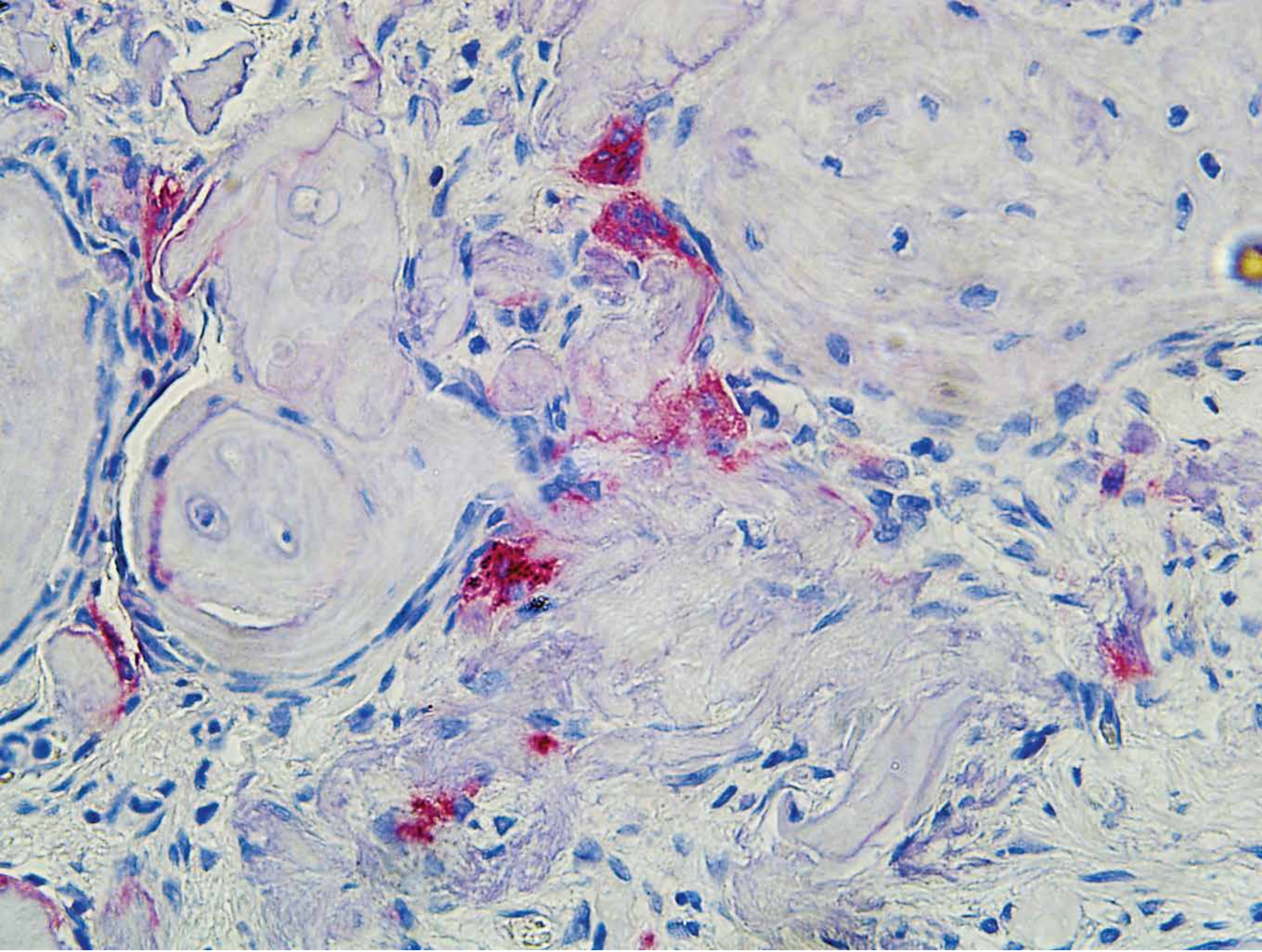

Supplement: S4 File — (PDF) [file pone.0344844.s004.pdf]

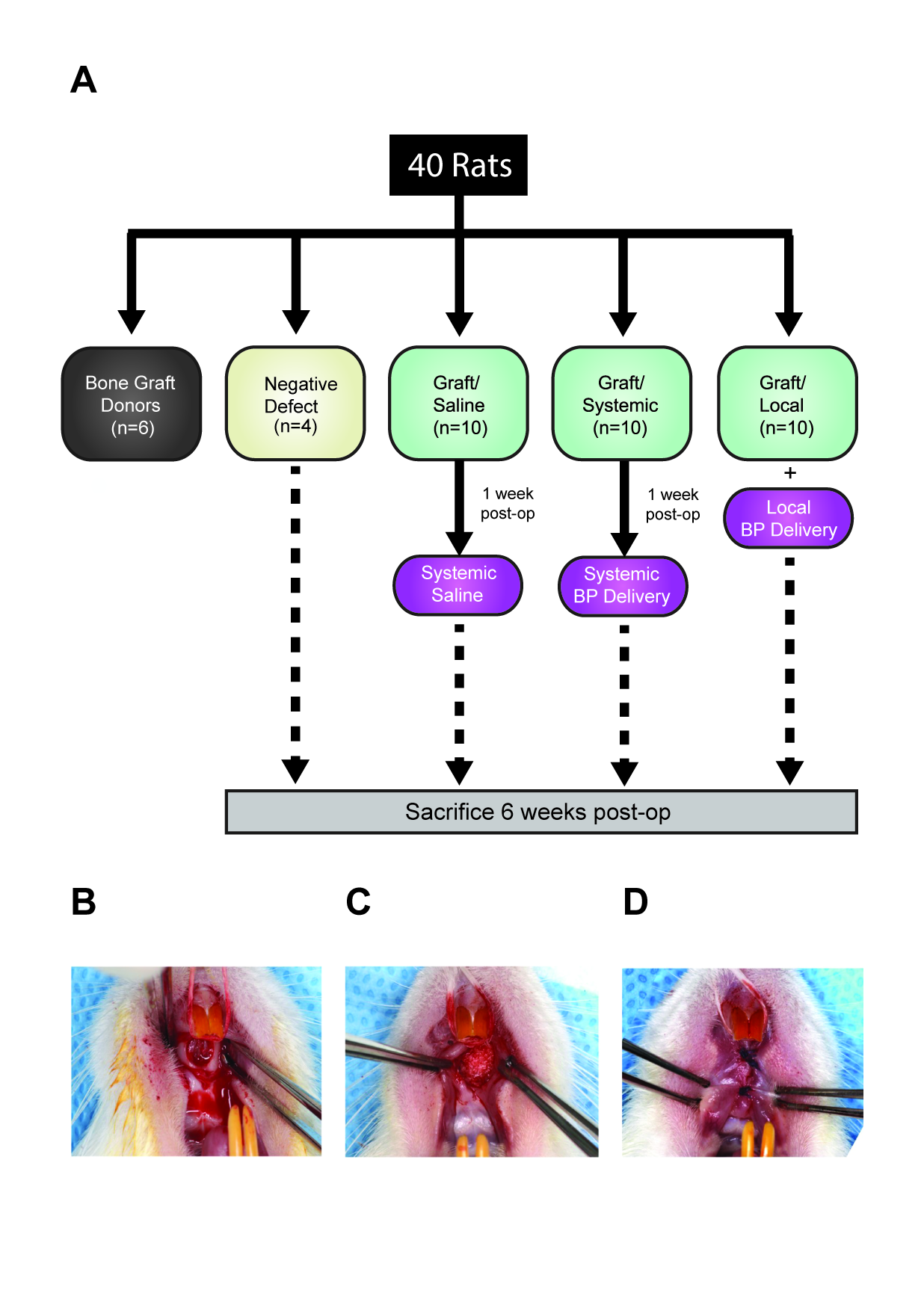

Supplement: S6 File — (TIF) [file pone.0344844.s006.tif]
